# Fibrinogen-like protein 2 aggravates nonalcoholic steatohepatitis via interaction with TLR4, eliciting inflammation in macrophages and inducing hepatic lipid metabolism disorder

**DOI:** 10.7150/thno.44297

**Published:** 2020-08-01

**Authors:** Junjian Hu, Hongwu Wang, Xitang Li, Yonggang Liu, Yuqiang Mi, Hongyan Kong, Dong Xi, Weiming Yan, Xiaoping Luo, Qin Ning, Xiaojing Wang

**Affiliations:** 1 Department and institute of infectious diseases, Tongji Hospital, Tongji Medical College, Huazhong University of Science and Technology, Wuhan, Hubei, China.; 2 Tianjin Second People's Hospital and Tianjin Institute of Hepatology, Tianjin, China.; 3 Department and institute of Pediatrics, Tongji Hospital, Tongji Medical College, Huazhong University of Science and Technology, Wuhan, Hubei, China.

**Keywords:** Fibrinogen-like protein 2, Nonalcoholic steatohepatitis, Macrophage, Toll-like receptor 4, Lipid metabolism

## Abstract

**Rationale:** The functions of fibrinogen-like protein 2 (fgl2) have been studied in many inflammatory and neoplastic diseases, but the role of fgl2 in nonalcoholic fatty liver disease has not yet been elucidated. In this study, we sought to investigate the role of fgl2 in the pathogenesis of nonalcoholic steatohepatitis (NASH).

**Methods:** Hepatic fgl2 expression was tested in patients with nonalcoholic fatty liver (NAFL) or NASH and controls. Wild-type and fgl2-/- C57BL/6 mice were subjected to a methionine/choline-deficient (MCD) diet or a high-fat diet (HFD) to establish NASH models. Bone marrow-derived macrophages (BMDMs) stimulated with LPS or free fatty acids were used for the *in vitro* study.

**Results:** In both humans and mice with NASH, macrophage accumulation was concomitant with significantly increased fgl2 expression in the liver. Fgl2 deficiency attenuated liver steatosis and inflammation in diet-induced murine models of NASH. In both liver tissues and BMDMs from NASH mice, fgl2 deficiency resulted in reduced levels of proinflammatory cytokines and reactive oxygen species (ROS) compared with levels in wild-type controls. Activation of NF-κB, p38-MAPK and NLRP3 inflammasomes was also suppressed upon fgl2 disruption. Moreover, lipogenic genes (Fasn and SREBP-2) were downregulated while lipolytic genes (PPAR and CPT1A) were upregulated in the livers of fgl2-/- NASH mice. Primary hepatocytes incubated with the medium collected from fgl2-/- BMDMs showed less fat deposition than those incubated with WT BMDMs. Furthermore, we discovered that fgl2 combined with TLR4 mediates the activation of the Myd88-dependent signaling pathway, which may contribute to inflammation and lipid metabolism disorders.

**Conclusions:** These data suggest that fgl2 aggravates the progression of NASH through activation of NF-κB, p38-MAPK and NLRP3 inflammasomes in macrophages, which consequently induces overproduction of proinflammatory cytokines and lipid metabolism disorders. An interaction of fgl2 and TLR4 may in part contribute to the activation of inflammatory signaling pathways in macrophages.

## Introduction

Nonalcoholic fatty liver disease (NAFLD) has become the most common chronic liver disease in the world, affecting up to 30% of the adult population 1. The NAFLD spectrum extends from simple hepatic steatosis to the concomitant presence of inflammation and ballooning, which define nonalcoholic steatohepatitis (NASH) 2. It can progress to increasing stages of fibrosis and ultimately cirrhosis and cirrhosis-related complications such as hepatocellular carcinoma (HCC) 3. The “two-hit” hypothesis has been considered the pathogenesis leading to liver injury, inflammation and fibrosis in NASH 4. This view has been challenged by the “multiple parallel hits” hypothesis in which different pathogenic events, such as lipotoxicity, oxidative stress, insulin resistance, and inflammation, occur in parallel to trigger disease development 5-8.

Hepatic macrophages, which comprise liver-resident Kupffer cells and recruited monocyte-derived macrophages, have been identified as one of the key mediators triggering the liver inflammatory response in NASH 9, 10. The accumulation and inflammatory polarization of hepatic macrophages is considered a hallmark feature of progressive disease in the liver of patients with NASH 11-13. Animal studies have also shown the infiltration of macrophages in the livers of methionine-choline-deficient (MCD) diet- and high-fat diet (HFD)-induced NASH 14. Depletion of hepatic macrophages or inactivation of proinflammatory mediators substantially blunted NASH development in murine models 9. However, the cellular mechanisms in the activation of hepatic macrophages during NASH development remain largely unknown.

Fibrinogen-like protein 2 (Fgl2), which belongs to the fibrinogen superfamily, can be expressed as a membrane-bound protein with coagulation activity or in a secreted form possessing unique immune suppressive functions. Membrane-bound fgl2 (mfgl2) is mainly expressed on the surface of macrophages 15 and endothelial cells 16, while soluble fgl2 (sfgl2) is predominantly produced by regulatory T cells 17. mFgl2 mediates macrophage activation 18, cell adhesion, trans-endothelial migration 19 and tumor growth 20 while sFgl2 inhibits the proliferation of effector T cells 21 and maintains the immunosuppressive activity of Tregs 22. Previous work from our group indicated that interference with fgl2 expression significantly increased the survival rate and alleviated liver injury in a murine model of fulminant hepatic failure 23, 24. Furthermore, mfgl2 is directly related to the progression of hepatic inflammation in patients with severe hepatitis B 25. Colaket et al. reported that plasma levels of fgl2 are significantly higher in patients with NASH than in healthy controls 26. However, the exact functional role of fgl2 in the NASH pathogenesis is still lacking. The latest research showed that fgl2 deficiency decreases macrophage infiltration and shifts macrophage phenotypes 27; thus, we speculate that fgl2 may influence the disease progression of NASH by regulating the function of macrophages.

In the present study, we sought to determine the contribution of fgl2 to the pathogenesis of diet-induced NASH in mice. We found that fgl2 interacted with TLR4 on macrophages and regulated inflammatory signaling pathways and the Nod-like receptor protein 3 (NLRP3) inflammasome, leading to liver damage and lipid metabolism disorders in the progression of NASH. Fgl2 may become a potential therapeutic target in NASH treatment.

## Materials and Methods

### Human liver samples

Liver biopsy sections that had been diagnosed with NAFL or NASH were obtained from Tianjin Second People's Hospital and Tianjin Institute of Hepatology (Tianjin, China). Human liver samples without NAFLD used as controls were obtained from the Department of Infectious Diseases, Tongji Hospital (Wuhan, Hubei, China). The study was approved by the Tianjin Second People's Hospital Ethics Committee and the Tongji Hospital Ethics Committee. General information regarding the patients is shown in Table S1.

### Animal experiment

Eight- to ten-week-old male mice were used in this study. Wild-type C57BL/6 mice were obtained from Vital River Laboratory Animal Technology (Beijing, China). Fibrinogen-like protein 2 knockout (fgl2-/-) mice were constructed by Shanghai Model Organisms Center, Inc. (Shanghai, China). Their age- and sex-matched homozygous wild-type littermates were used as controls. To establish NASH models, the mice were maintained on 12:12-hour light-dark cycles and fed the MCD diet (TP3001, TROPHIC Animal Feed High-tech Co., Ltd, China) for 4-6 weeks or HFD (TP23400, TROPHIC) for 24 weeks. The MCS diet (TP3001S, TROPHIC) or standard chow diet (Jiangsu Xietong Pharmaceutical Bioengineering Co., Ltd, China) were used as control diets. All mice were kept in the animal experiment center of Tongji Hospital, following the procedures approved by the Tongji Hospital Animal Ethics Committee. The main components of the MCD diet and high-fat diet are shown in Table S2 and Table S3. The expression of fgl2 in WT or fgl2-/- mouse livers was tested by immunohistochemistry and western blotting using monoclonal fgl2 antibody (H00010875-M01, Abnova, Taiwan) (Figure S1).

### Histological and immunohistochemistry analysis

Human or mouse liver samples were fixed in 4% paraformaldehyde for 24 hours, embedded in paraffin and cut into sections of 4 μm thickness. After hematoxylin and eosin staining, the NAFLD activity score (NAS) was used to quantify lobular inflammation, ballooning and steatosis in patients and murine models of NASH. To detect the expression of CD68, F4/80 and fgl2, paraffin-embedded human or mouse liver blocks were stained with the following antibodies: CD68 (1:200) (ab201340, Abcam, UK), F4/80 (1:100) (70076, CST, USA), and fgl2 (1:100) (H00010875-M01, Abnova, Taiwan). The sections were observed under a microscope (CX22, OLYMPUS, Japan).

### Immunofluorescence analysis

Tissue paraffin sections were dewaxed and subjected to antigen retrieval treatment. After washing and blocking, the slides were incubated with the following primary antibodies: CD68 (1:200) (ab213363, Abcam, UK), fgl2 (1:200) (H00010875-M01, Abnova, Taiwan), and F4/80 (1:200) (ab6640, Abcam, UK). The subsequent secondary antibodies were Cy3-conjugated AffiniPure goat anti-rat IgG (1:200) (GB21302, Servicebio, China) and fluorescein isothiocyanate-labeled goat anti-mouse IgG (1:200) (GB22301, Servicebio, China). DAPI (G1012, Servicebio, China) was used for nuclear staining. The sections were observed under a fluorescence microscope (BX53, OLYMPUS, Japan). Three sections and 3-5 different positive microscopic fields in each section were chosen for semiquantitative analysis. Fluorescence intensity was analyzed by ImageJ software.

### Western blot analysis

Proteins in mouse liver and BMDMs were extracted using RIPA lysis buffer (AR0102, BOSTER, China) and nuclear/cytoplasmic extraction reagents (78833, Thermo Scientific, USA). Protein samples were resolved on polyacrylamide gels made by NewFlash Protein AnyKD PAGE (8012011, Biosci, Dakewe Biotech, China) and transferred onto PVDF membranes. Membranes were blocked with 5% milk and incubated with primary antibodies and secondary antibodies. Immunoreactivity was tested by an enhanced chemiluminescence system (ChemiDoc XRS+, Bio-Rad, USA). Densitometry was performed using ImageLab software. Relative quantification was achieved from three independent experiments, and at least six blots from each group were used for statistical analysis. The antibodies were as follows: NF-κB p65 (1:1000) (8284, CST, USA), phospho-NF-κB p65 (1:1000) (3039, CST, USA), p38-MAPK (1:1000) (8690, CST, USA), phospho-p38-MAPK (1:1000) (4511, CST, USA), β-actin (1:2000) (4970, CST, USA), histone H3 (1:1000) (4499, CST, USA), NLRP3 (1:1000) (15101, CST, USA), IL-1β (1:1000) (12507, CST, USA), pro-Caspase+p10 (1:1000) (ab179515, Abcam, UK), IL-18 (1:1000) (BA14935, BOSTER, China), TLR4 (1:1000) (ab13556, Abcam, UK), MyD88 (1:800) (66660-1-Ig, Proteintech, USA), TRAF6 (1:800) (66498-1-Ig, Proteintech, USA), fgl2 (1:1000) (H00010875-M01, Abnova, Taiwan), HRP goat anti-rabbit IgG (1:5000) (A21020, Abbkine, USA), and HRP goat anti-mouse IgG (1:5000) (A21010, Abbkine, USA).

### Cytokine enzyme-linked immunosorbent assay (ELISA)

Cytokines in liver homogenates from the mice and the culture supernatants of BMDMs were determined by ELISA kits (TNF-α 1217202, IL-1β 1210122, MCP-11217392, IL-6 1210602, Dakewe Biotech, China; IL-18 70-EK218-96, MutiSciences, China) according to the manufacturers' instructions.

### Quantitative real-time PCR

Total RNA was extracted from liver tissues using TRIzol reagent (15596026, Invitrogen, USA). The purity and concentration of RNA were determined from the OD 260/280 readings using a spectrophotometer (NanoDrop One, Thermo, USA), and the integrity of the RNA was detected by agarose gel electrophoresis. Then, total RNA was reserve-transcribed into cDNA with a ReverTra Ace qPCR RT Kit (FSQ-101, TOYOBO, Japan) in an RNase-free environment, and gradient PCR was used to achieve the best annealing temperature. Gene expression was detected by a real-time PCR system (CXF96, Bio-Rad, USA) using SYBR Green Real-time PCR Master Mix (QPK-201, TOYOBO, Japan). Primer sequences used were as follows: Fgl2 forward: 5'-GCCAAATGTGAGTCCCTGGAA-3', reverse: 5'-TTCCACCCAAGAGCACGTTTAAG-3'; F4/80 forward: 5'-ACCACAATACCTACATGCACC-3', reverse: 5'-AAGCAGGCGAGGAAAAGATAG-3'; Clec4f forward: 5'-CCTGAGTGGAATAAAGAGCCTC-3', reverse: TCCTCATAGTCCCTAAGCCTC; Fasn forward: 5'-GGAGGTGGTGATAGCCGGTAT-3', reverse: 5'-TGGGTAATCCATAGAGCCCAG-3'; SREBP-2 forward: 5'-ACAGACACAAGGGCTAGGCT-3', reverse: 5'-GTGCTTCAACCCCACCTACT-3'; PPARα forward: 5'-AGAGCCCCATCTGTCCTCTC-3', reverse: 5'-ACTGGTAGTCTGCAAAACCAAA-3'; and CPT1A forward: 5'-CTCCGCCTGAGCCATGAAG-3', reverse: 5'-CACCAGTGATGATGCCATTCT-3'.

### Flow cytometry analysis

Liver tissue was digested into a single cell suspension by injecting collagenase IV (C8160, Solarbio, Beijing) via the portal vein. Cells were resuspended in 40% Percoll (17-0891-09, GE Healthcare, Sweden) for Percoll gradient centrifugation at 800g to obtain hepatic nonparenchymal cells. The cells were aliquoted to 1 × 10^6^ cells/100 μl in FACS tubes and blocked with purified CD16/32 antibody (101302, BioLegend, USA). Cells were then stained with PerCP/Cyanine5.5-anti-mouse CD11b (101227, BioLegend, USA), FITC-anti-mouse CD45 (103108, BioLegend, USA), APC-anti-F4/80 (123116, BioLegend, USA) and PE-anti-CD284 (TLR4) (145403, BioLegend, USA) antibodies. Fgl2 monoclonal antibody (H00010875-M01, Abnova, Taiwan) was used as the primary antibody, and PE-goat-anti-mouse IgG (405370, BioLegend, USA) was used as the secondary antibody. Corresponding isotype antibodies were used as controls. The cells in the CD45+CD11b+F4/80+ gate were considered macrophages. The samples were sorted on an LSRFortessa cytometer, and data were analyzed with FlowJo-X software.

### Cell culture and treatment

Bone marrow cells were flushed from the femur and tibia by ice-cold sterile PBS in a sterile environment. RBC lysis buffer (BSA06M1, BioFlux, Romania) was used to remove erythrocytes. The cells were cultured in 6-well culture plates with DMEM containing 10% FBS. Then, 20 ng/ml M-CSF (576404, BioLegend, USA) was added to the culture for 7 days to obtain BMDMs as previously described 28. Before stimulation, cell viability was checked by trypan blue staining (T10282, Invitrogen, USA). BMDMs were stimulated by LPS (100 ng/ml) for 6 hours or FFA (800 μmol/L), which was prepared by oleic acid (O1008, Sigma-Aldrich, USA) and palmitic acid (P5585, Sigma-Aldrich, USA) at a 2:1 ratio, for 24 hours.

Primary hepatocytes were isolated from WT C57BL/6J mice as previously described 29. After attachment, hepatocytes were further incubated in DMEM containing 10% FBS, 100 U/ml penicillin and 100 μg/ml streptomycin for 24 hours. Then, primary hepatocytes were incubated in DMEM mixed with conditioned medium from WT or fgl2-/- BMDMs that had been treated with 100 ng/ml LPS or 800 μmol/L FFA at a 1:1 ratio for 24 hours. The hepatocytes were stained by oil red O and quantified for fat content by ImageJ software. Expression of lipid metabolism genes was also detected.

### Macrophage depletion and adoptive transfer

Macrophage depletion was performed according to the manufacturer's instructions with an intraperitoneal injection of 150 μl of Clodronate Liposomes (F70101C-A-10, FormuMax, USA) twice a week for 3 weeks during MCD diet feeding. Effectivity of macrophage depletion was evaluated by immunohistochemistry (Figure S5A). A total of 5 × 10^5^ WT or fgl2-/- BMDMs were rapidly injected into WT mice through the caudal vein. Vehicle (PBS) injection was used as control. Liver function markers and cytokines in liver tissues were tested in week 4.

### Lentivirus transduction

HBLV-h-Fgl2-GFP-PURO and HBLV-GFP-PURO as controls were constructed by Hanbio Biotech Co., Ltd. (Shanghai, China). THP-1 cells were cultured in 1640 medium containing 10% FBS in 12-well culture plates at 1 × 10^5^ cells/ml, and then THP-1 cells were infected with HBLV-h-Fgl2-GFP-PURO and HBLV-GFP-PURO at a multiplicity of infection of 100. The cells were harvested at 96 hours post infection, and the expression of fgl2, TLR4, MyD88 and TRAF6 was tested by western blotting. The cytokine levels in culture supernatants were determined by ELISA (TNF-α 1117202, IL-1β 1110122, and IL-6 1110602, Dakewe Biotech, China).

### Coimmunoprecipitation

THP-1 cells were cultured in 6-well culture plates and differentiated into mature macrophages by PMA for 24 hours. The cells were then stimulated with LPS (100 ng/ml) or FFA (800 μmol/L) for 24 hours. Total proteins were extracted by cell lysis buffer for western blotting and IP (P0013, Beyotime, China). The proteins were incubated with antibodies in EP tubes at 4°C overnight. An IP & CoIP Kit (abs955, Absin, China) was used to obtain precipitated proteins. The results were validated by western blotting. TLR4 polyclonal antibody (PAB0374, Abnova, Taiwan) and fgl2 antibody (H00010875-M01, Abnova, Taiwan) were used as target antibodies. Rabbit IgG (B900610, Proteintech, USA) and mouse IgG (ab190475, Abcam, UK) were used as control antibodies.

### Statistics

Data are expressed as the means ± standard deviation (SD). Unpaired two-tailed Student's t tests were used for comparisons between two experimental groups when data conformed to a normal distribution and homogeneity of variance. The Mann-Whitney U test was used for nonnormally distributed data. Data with a normal distribution and homogeneity of variance from multiple groups were compared using one-way ANOVA with Bonferroni correction or two-way ANOVA. Two-sided P-values of less than 0.05 were considered statistically significant. Spearman's rank correlation coefficient analysis was applied to analyze the correlation between fgl2 expression and NAFLD activity score. All statistical analyses were performed using SPSS (Statistical Package for the Social Sciences) version 25.0 software (SPSS Inc.), and figures were plotted by GraphPad Prism 6.0. The number of animals or measurements in each group is indicated in the figure legends. All experiments were repeated independently at least three times.

For further details regarding the materials used, please refer to the Supplementary materials and methods.

## Results

### Fgl2 expression was significantly increased concomitantly with macrophage accumulation in the livers of patients with NASH

**Figure 1:**
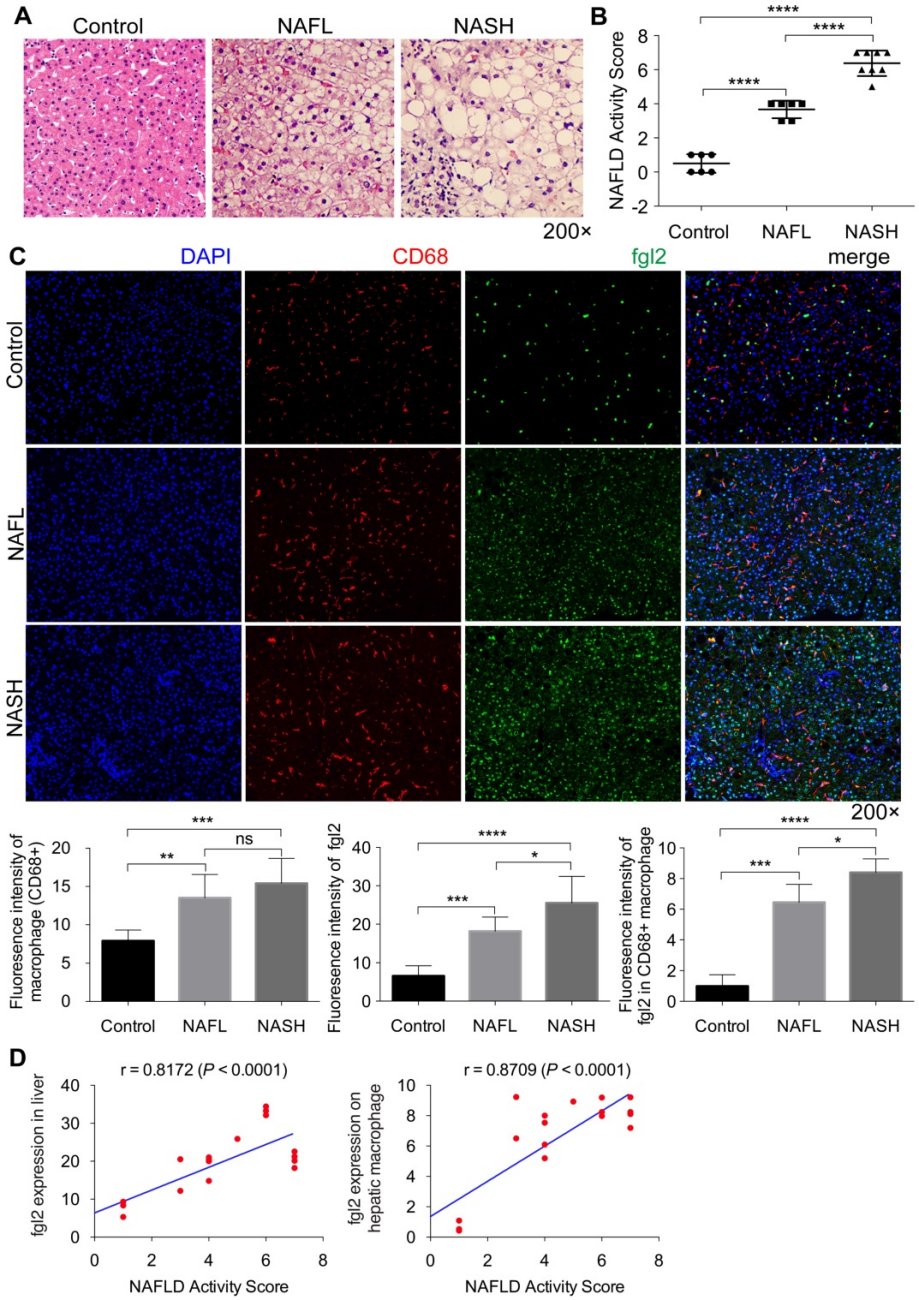
** Fgl2 expression was enhanced concomitantly with hepatic macrophage accumulation in patients with NASH.** Liver sections of patients with NAFL and NASH and controls (human subjects without NAFLD) were stained with HE (A). CD68+ macrophages (red) and fgl2 (green) were observed by immunofluorescent staining (C). The NAFLD active score was evaluated in controls (n=6), NAFL (n=6) and NASH (n=8) human subjects (B). The fluorescence intensity of fgl2-, CD68- and fgl2-positive macrophages (fgl2 in CD68+) was evaluated by ImageJ software (C). Correlations between fgl2 expression in liver/hepatic macrophages and NAFLD activity score were also analyzed (D). Five microscopic fields per liver section from 3 patients in each group were counted. The data represent the mean ± SD from at least three independent experiments. Statistical differences were determined by one-way ANOVA with Bonferroni correction and Spearman's rank correlation coefficient analysis was applied to analyze the correlation between fgl2 expression levels and NAFLD activity score. *P<0.05, **P<0.01, ***P<0.001, ****P<0.0001; ns, not significant.

To address the relevance of fgl2 and human NASH, we first examined fgl2 expression in liver sections of human subjects. Compared with the control, liver sections of patients with nonalcoholic fatty liver (NAFL) and NASH revealed increased fat deposition, and patients with NASH showed significant hepatic inflammatory injury, as indicated by HE staining and the NAFLD activity score (Figure 1A, B). Immunohistochemistry and immunofluorescence staining were used to detect the expression of hepatic fgl2 and macrophages, which are major sources of fgl2 under pathological conditions. The colocalization of fgl2 and macrophages was observed particularly in the liver of patients with NASH. The number of CD68+ macrophages and the expression of fgl2 were both increased in patients with NAFL and NASH compared with controls, and patients with NASH exhibited higher expression of fgl2 than patients with NAFL (Figure 1C, Figure S2A). Moreover, the number of fgl2-positive macrophages in the liver sections of patients with NASH was greater than that in patients with or without NAFLD (Figure 1C). The expression of fgl2 in liver and hepatic macrophage were positively correlated with the NAFLD activity score (Figure 1D). These data suggested that fgl2 may be involved in the progression of NASH.

### The accumulated hepatic macrophages revealed a gradual increase in fgl2 expression with the progression of NASH in mice

**Figure 2:**
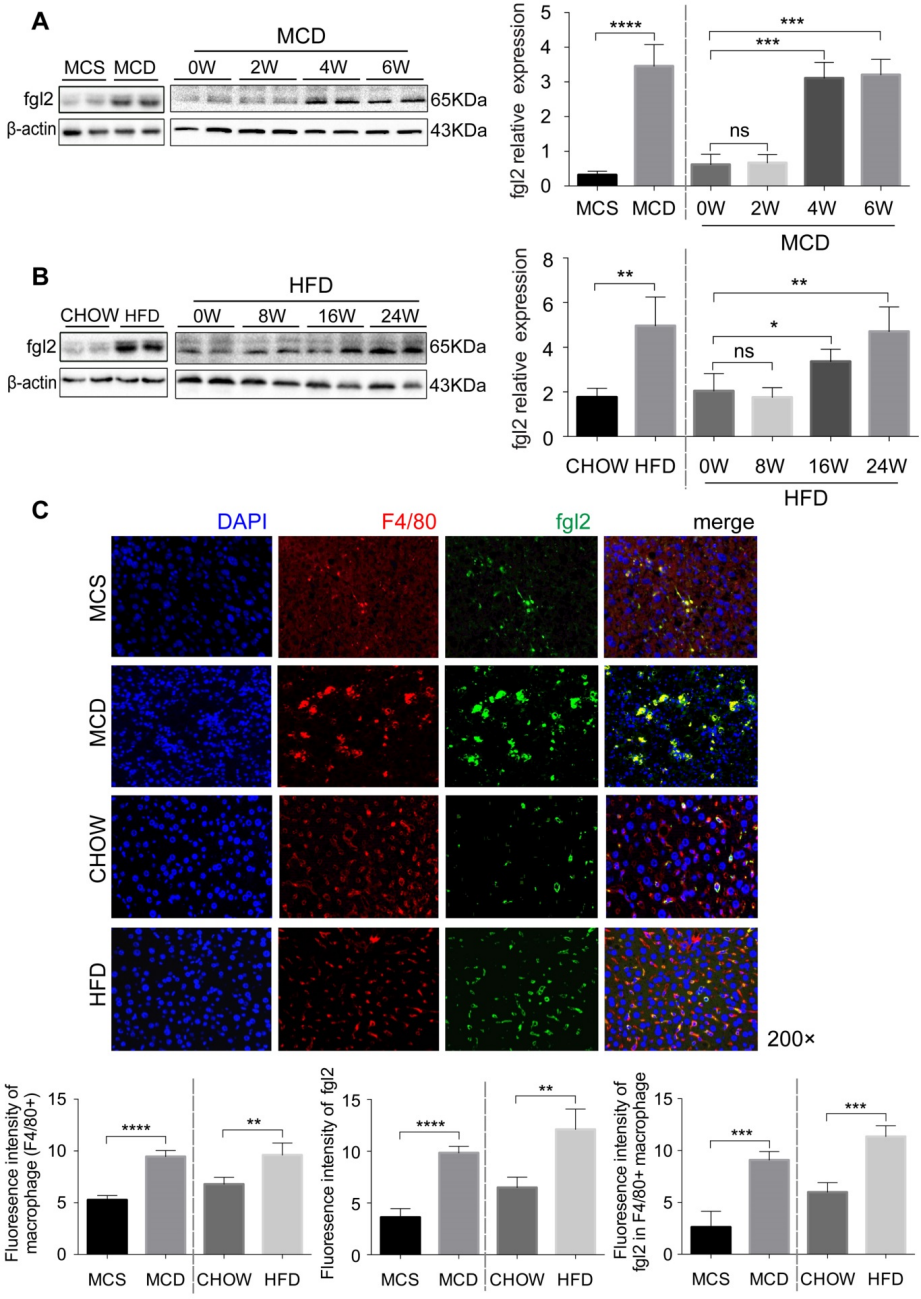
** The accumulated hepatic macrophages showed increased expression of fgl2 in the livers of NASH mice.** Mice were subjected to an MCD diet (A) for 6 weeks and HFD (B) for 24 weeks to establish NASH models. The MCS diet (A) and chow diet (B) were used as controls. Mice were sacrificed at the indicated time points. Hepatic fgl2 expression was analyzed by western blotting (A, B). For bar graphs, n=6-10 in each group. F4/80+ macrophages (red) and fgl2 (green) were observed by immunofluorescent staining (C). Five microscopic fields per liver section from 3 animals in each group were counted. The data represent the mean ± SD from at least three independent experiments. For multiple group comparisons, significant differences were determined by one-way ANOVA with Bonferroni correction. Differences between two experimental groups were determined by unpaired two-tailed Student's t-test. *P<0.05, **P<0.01, ***P<0.001, ****P<0.0001; ns, not significant.

To further investigate the role of fgl2 in NASH, we fed C57BL6/J mice an MCD diet for 6 weeks or a HFD for 24 weeks to establish a mouse model of NASH. Both MCD-fed and HFD-fed mice had higher expression of fgl2 in the liver than controls (Figure 2A, Figure S2B). Unlike MCD-fed mice, in which the liver fgl2 level was increased since week 4, HFD-fed mice started to exhibit enhanced liver fgl2 expression after week 16 (Figure 2B). In MCD-fed mice, the number of F4/80+ macrophages began to increase at week 2 and peaked between weeks 3 and 4, while in HFD-fed mice, macrophages continued to accumulate throughout the feeding process (Figure S3A, B). Similar to patients with NASH, colocalization of fgl2 and F4/80+ macrophages was detected in the livers of NASH mice. Meanwhile, fgl2 expression on macrophages in NASH mice was higher than that in control mice (Figure 2C, Figure S2E). Together, these data indicated that the upregulated expression of fgl2 in the liver paralleled the accumulation of macrophages in diet-induced mouse NASH models.

### Fgl2 deficiency attenuated liver inflammatory injury in NASH mice

**Figure 3:**
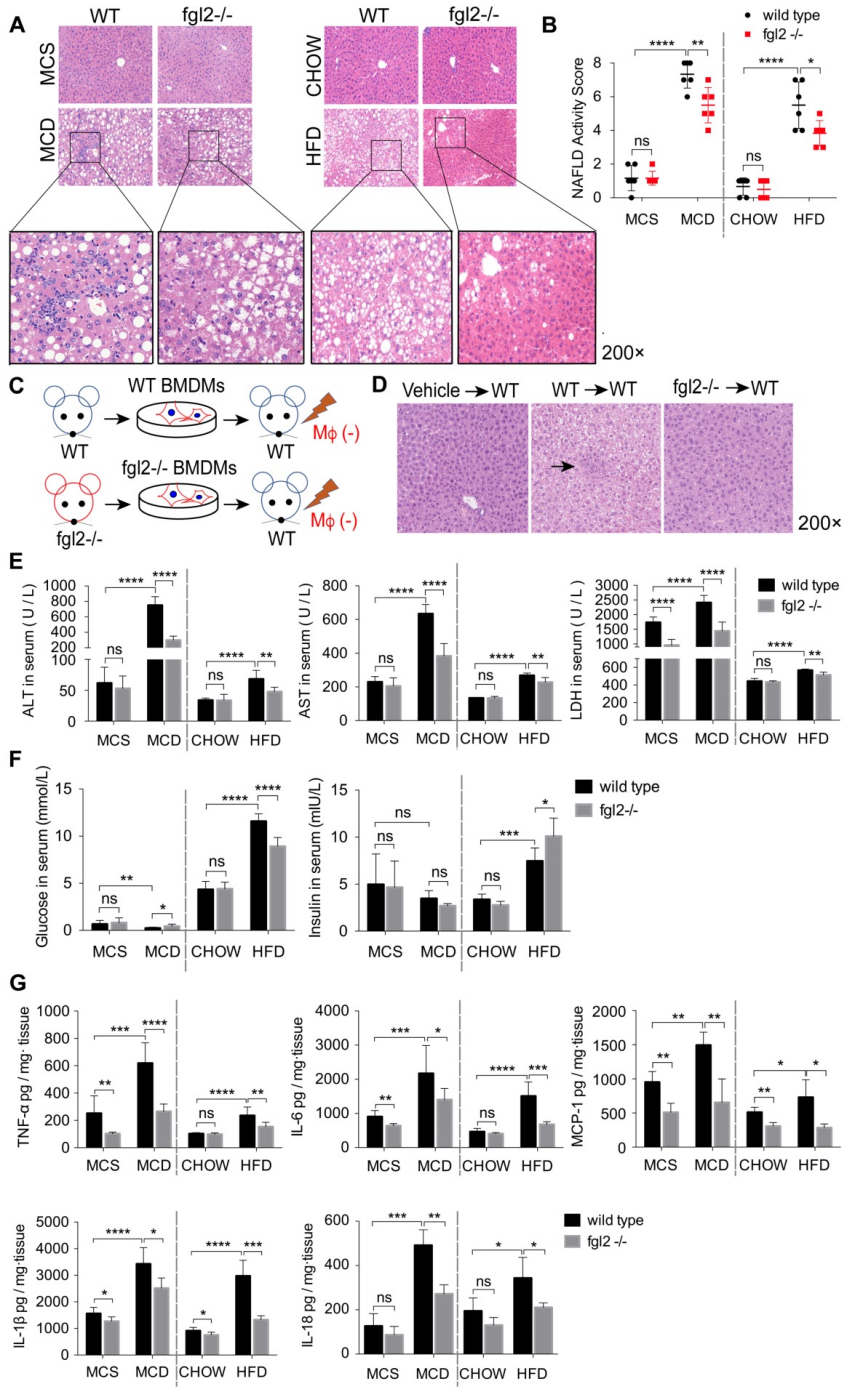
** Fgl2 deficiency attenuated liver inflammatory injury in NASH mice.** In MCD-fed or HFD-fed WT and fgl2-/- mice, HE staining was performed to detect histological changes in the liver (A). The NAFLD activity score was evaluated (B). BMDMs were isolated from WT or fgl2-/- mice and injected into macrophage-depleted WT NASH mice (C). Histological changes were detected by HE staining (D, arrows indicate inflammatory infiltration). Serum ALT, AST, LDH (E) and fasting glucose (F) were tested by an automatic biochemical analyzer (n=10 in each group). The levels of serum insulin were tested by an ELISA kit (F). The levels of the proinflammatory cytokines TNF-α, MCP-1, IL-6, IL-1β and IL-18 in the liver were tested by ELISAs (G). For bar graphs, n=6-10 in each group. The data represent the mean ± SD from at least three independent experiments. Statistical differences were determined by two-way ANOVA. *P<0.05, **P<0.01, ***P<0.001, ****P<0.0001; ns, not significant.

To evaluate the effects of fgl2 disruption on diet-induced steatohepatitis, we examined the liver histologic changes by HE staining in MCD-fed or HFD-fed WT and fgl2-/- mice. In MCD-fed and HFD-fed WT mice, the livers presented extensive lipid accumulation, with varying degrees of inflammatory cell infiltration and hepatocellular ballooning. MCD-induced NASH showed more severe lobular and portal inflammation. The disruption of fgl2 attenuated inflammatory injury in both NASH models, as evidenced by the reductions in inflammatory cell infiltration and hepatocyte ballooning (Figure 3A). The fgl2-/- mice also showed lower NAFLD activity scores than WT mice in these two models (Figure 3B). Accordingly, fgl2 deficiency resulted in notable decreases in ALT, AST and LDH levels (Figure 3E), indicating its protective role. HFD-fed WT mice showed higher body weights than chow-fed controls, while fgl2 deficiency markedly decreased their weight gains before 16 weeks (Figure S4B). Consistent with previous studies 30, the body weights of MCD-fed mice were reduced within the feeding time. No difference was found in body weights between MCD-fed fgl2-/-mice and WT mice (Figure S4A). Upon fgl2 disruption, the level of fasting serum glucose was decreased in HFD-fed mice but increased in MCD-fed mice. The serum insulin level was increased in fgl2-/- HFD-fed mice, but no difference was found in MCD-fed mice (Figure 3F). Glucose tolerance test (GGT) and insulin tolerance test (ITT) demonstrated that insulin resistance could be improved when fgl2 was deficient (Figure S4C, D). Importantly, hepatic expression of proinflammatory cytokines, including TNF-α, IL-6, MCP-1, IL-1β and IL-18 (Figure 3G), as well as ROS production in liver and hepatic macrophages (Figure S6A-D), were all markedly inhibited after fgl2 disruption. To further clarify the specific role of macrophage-expressed fgl2 in NASH, we isolated BMDMs from WT or fgl2-/- mice and adoptively transferred them into macrophage-depleted MCD-fed WT mice (Figure 3C). After macrophage depletion, inflammatory injury and lipid deposition were significantly alleviated in the livers of MCD-fed mice. Compared with fgl2-/- BMDMs, WT BMDMs injected into macrophage-exhausted mice led to more severe liver injury (Figure 3D, S5B) and higher levels of ALT, AST (Figure S5C) and proinflammatory cytokines (Figure S5D). These data strongly support the important role of macrophage-expressed fgl2 in the progression of NASH.

### Fgl2 deficiency ameliorated liver steatosis in HFD-induced NASH by regulating lipid metabolism

**Figure 4:**
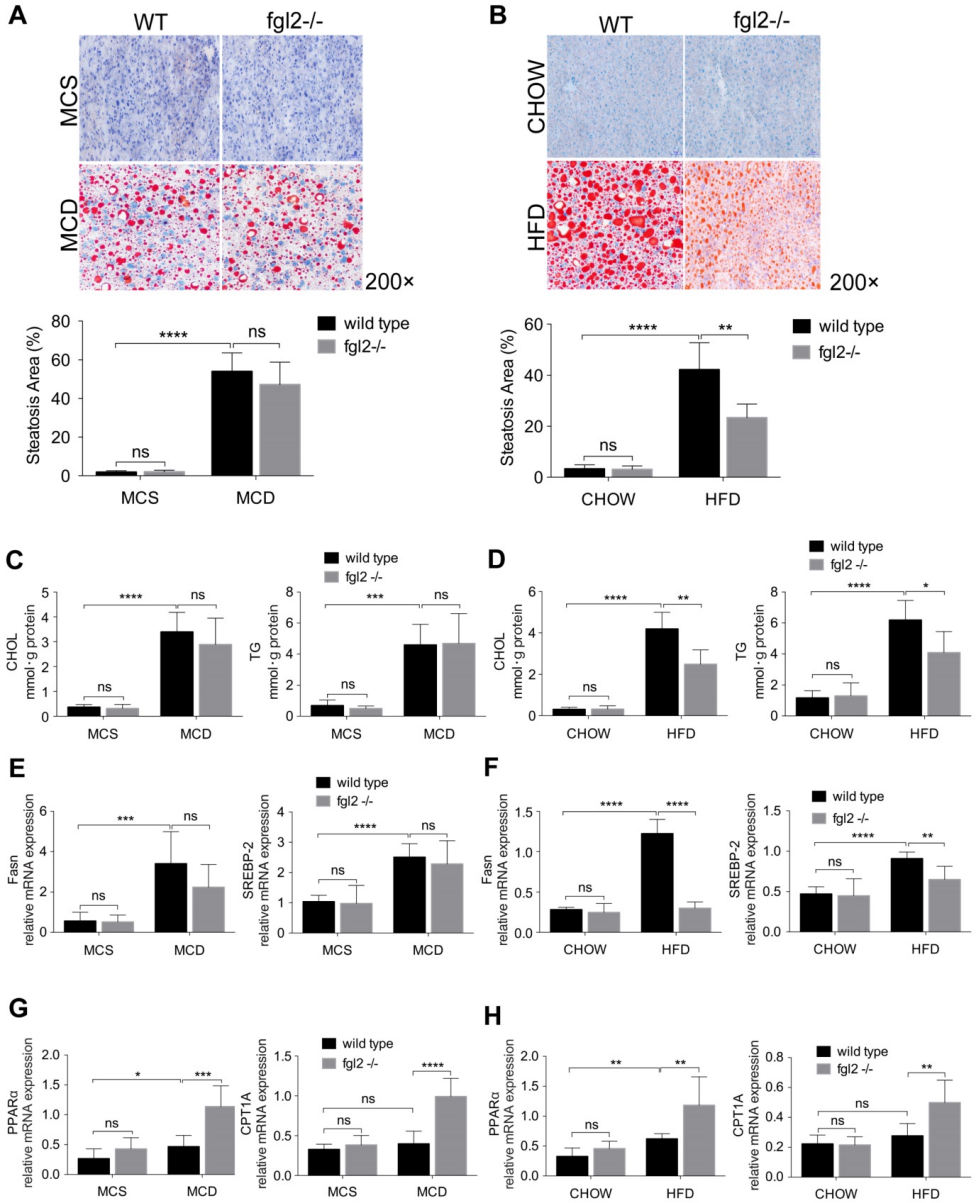
** Fgl2 deficiency ameliorated liver steatosis in HFD-induced NASH by regulating lipid metabolism.** In MCD-fed (A) or HFD-fed (B) WT and fgl2-/-mice, oil red O staining was performed to detect liver steatosis. The levels of cholesterol and triglycerides in the liver were examined (C, D). Hepatic mRNA levels of genes involved in lipogenesis (Fasn, SREBP-2) (E, F) or lipolysis (PPARα, CPT1A) (G, H) were tested by real-time PCR. For bar graphs, n=8 in each group. The data represent the mean ± SD from at least three independent experiments. Statistical differences were determined by two-way ANOVA. *P<0.05, **P<0.01, ***P<0.001, ****P<0.0001; ns, not significant.

To determine the contribution of fgl2 to liver steatosis in NASH, we examined fat deposition in mouse livers by oil red O staining. We only observed a notable reduction in liver fat deposition in HFD-fed (Figure 4B) but not MCD-fed fgl2-/- mice (Figure 4A). Consistent with this finding, cholesterol and triglyceride levels decreased in HFD-fed fgl2-/- mice (Figure 4D) but barely changed in MCD-fed fgl2-/- mice (Figure 4C). To identify the lipid metabolism pathways involved in this process, we further detected genes related to cholesterol and triglyceride metabolism. The expression of Fasn and SREBP-2, which are key components in triglyceride and cholesterol synthesis, was decreased in HFD-fed fgl2-/- mice compared with WT control mice (Figure 4F). However, in MCD-fed fgl2-/- mice, Fasn and SREBP-2 had no evident changes compared with controls (Figure 4E). The expression of PPARα and CPT1A, which belong to a key pathway of lipolysis, was elevated in fgl2-disrupted NASH models (Figure 4G, H). These data demonstrated that fgl2 upregulated Fasn and SREBP-2, which contribute to lipogenesis, but downregulated PPARα and CPT1A, which enhance lipolysis, particularly in HFD-induced NASH.

### Fgl2 promoted the production of proinflammatory cytokines in macrophages and subsequently influenced lipid metabolism in hepatocytes

**Figure 5:**
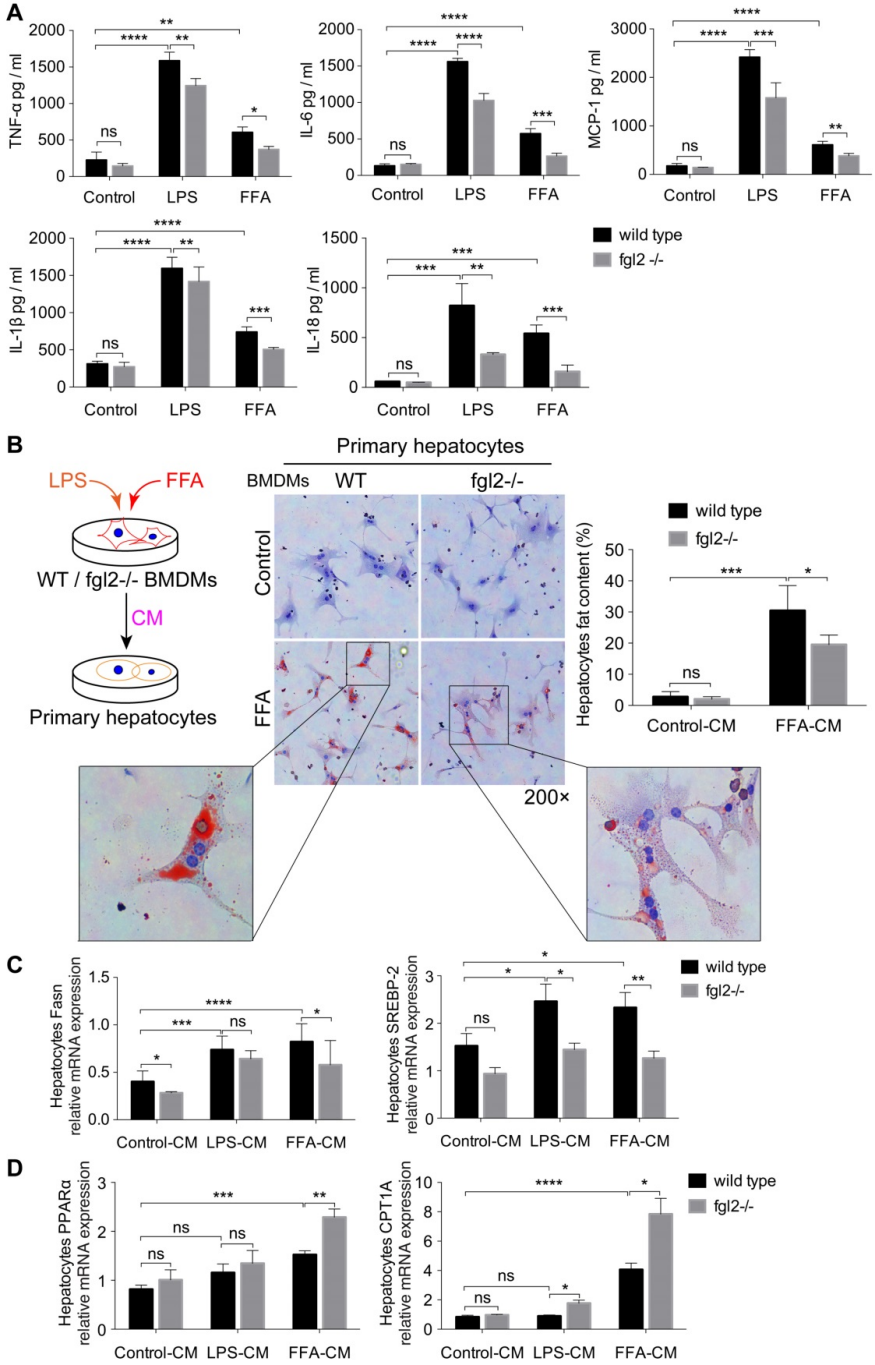
** Fgl2 deficiency reduced lipid accumulation in hepatocytes by inhibiting the secretion of proinflammatory cytokines in macrophages.** BMDMs from WT and fgl2-/- mice were stimulated with LPS (100 ng/ml) or FFA (800 μmol/L). The levels of proinflammatory cytokines, including TNF-α, MCP-1, IL-6, IL-1β and IL-18, in the supernatant of cell cultures were tested by ELISAs (A). Primary hepatocytes were isolated from C57BL/6J mouse livers and incubated with LPS- or FFA-BMDM-CM for 24 hours. The brief experimental procedure is shown in a diagram. Oil red O staining was used to detect fat deposition in primary hepatocytes after treatment with BMDM-CM (B). Then, the mRNA levels of genes involved in lipogenesis (Fasn, SREBP-2) (C) or lipolysis (PPARα, CPT1A) (D) in primary hepatocytes were tested by real-time PCR. For bar graphs, n=6 in each group. The data represent the mean ± SD from at least three independent experiments. Statistical differences were determined by two-way ANOVA. *P<0.05, **P<0.01, ***P<0.001, ****P<0.0001; ns, not significant.

To assess the influence of fgl2 on macrophages and clarify how fgl2 mediates hepatic lipid metabolism disorders, we first examined the levels of proinflammatory cytokines, including TNF-α, IL-6, MCP-1, IL-1β and IL-18. In LPS- or FFA-treated WT BMDMs, the levels of these cytokines were all elevated compared with those in control cells. Fgl2 disruption evidently downregulated the expression of these cytokines in BMDMs treated with LPS or FFA (Figure 5A). Meanwhile, the activated ROS produced by stimulated BMDMs also showed a reduction upon fgl2 disruption (Figure S6E). Since fgl2 is not expressed on hepatocytes 31, we hypothesized that the influence of fgl2 on hepatic lipid metabolism was mediated by macrophages. Thus, we incubated WT primary hepatocytes with conditioned medium (CM) from LPS- or FFA-treated WT and fgl2-/- BMDMs (Figure 5B). Interestingly, fat deposition decreased notably in hepatocytes incubated with fgl2-/- FFA-CM compared with those incubated with WT FFA-CM (Figure 5B). Then, we detected the expression of genes related to lipid metabolism. With regard to lipogenic genes, SREBP-2 expression was downregulated in hepatocytes cultured in fgl2-/- LPS-CM or fgl2-/- FFA-CM, while Fasn expression was only decreased in the fgl2-/- FFA-CM group (Figure 5C). For lipolytic genes, CPT1A was upregulated in both the fgl2-/- LPS- and FFA-CM groups, but PPARα was only increased in the fgl2-/- FFA-CM group (Figure 5D). Together, these data demonstrated that fgl2 promoted LPS/FFA-triggered macrophages to produce inflammatory cytokines, which further induced lipid metabolism disorder in hepatocytes.

### Fgl2 disruption suppressed activation of NF-κB and p38-MAPK signaling pathways in NASH

**Figure 6:**
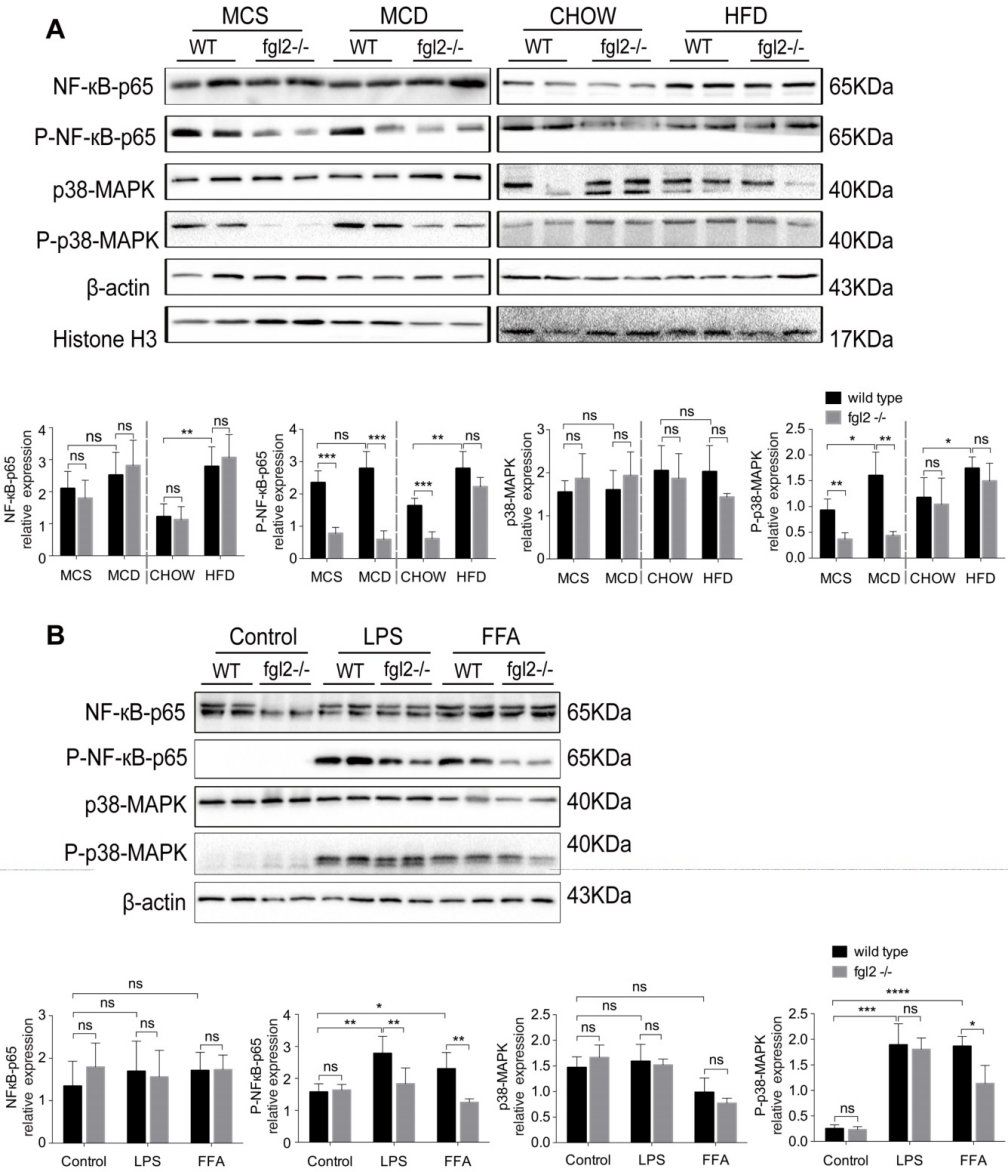
** Fgl2 disruption suppressed activation of the NF-κB and p38-MAPK signaling pathways in NASH.** For *in vivo* examination, the total protein was obtained from liver tissues of MCD-fed or HFD-fed WT and fgl2-/- mice. MCS-fed and chow-fed mice were used as controls. NF-κB-p65, p38-MAPK and their phosphorylated forms were analyzed by western blotting (n=6) (A). *In vitro*, BMDMs from WT or fgl2-/- mice were stimulated with LPS or FFA and tested for the normal and phosphorylated levels of NF-κB and p38-MAPK by western blotting (B). Image density was quantified using ImageLab software. For bar graphs, n=6 in each group. The data represent the mean ± SD from at least three independent experiments. Statistical differences were determined by two-way ANOVA. *P<0.05, **P<0.01, ***P<0.001, ****P<0.0001; ns, not significant.

Since fgl2 deficiency reduced the levels of proinflammatory cytokines in both MCD-fed and HFD-fed mice, we attempted to explore the potential signaling pathways involved. Previous studies have shown that NF-κB and MAPK signaling are key regulators of proinflammatory cytokines such as TNF-α, IL-1β 32, IL-6 33, and MCP-1 34. Thus, we detected the activation of NF-κB-p65, c-Jun N-terminal kinase (JNK) and p38-MAPK in diet-induced fgl2-/- and WT NASH models. In the two WT NASH models, the phosphorylation levels of NF-κB-p65, p38-MAPK (Figure 6A) and JNK (Figure S7A, B) were higher than in controls. Fgl2 disruption inhibited the phosphorylation of NF-κB-p65 and p38-MAPK levels in MCD-fed mice but not in HFD-fed mice (Figure 6A). JNK phosphorylation showed no significant alteration upon fgl2 disruption (Figure S7A, B). Similar results were observed *in vitro*. In BMDMs stimulated with FFA, both NF-κB-p65 and p38-MAPK phosphorylation levels were decreased in the fgl2-/- group compared with the WT group. However, only the NF-κB-p65 phosphorylation level was decreased in the fgl2-/- group when stimulated with LPS (Figure 6B). In brief, these results suggested that activation of NF-κB and p38-MAPK is critical for fgl2 to induce the production of proinflammatory cytokines.

### Fgl2 disruption inhibited NLRP3 inflammasome activation in NASH

**Figure 7:**
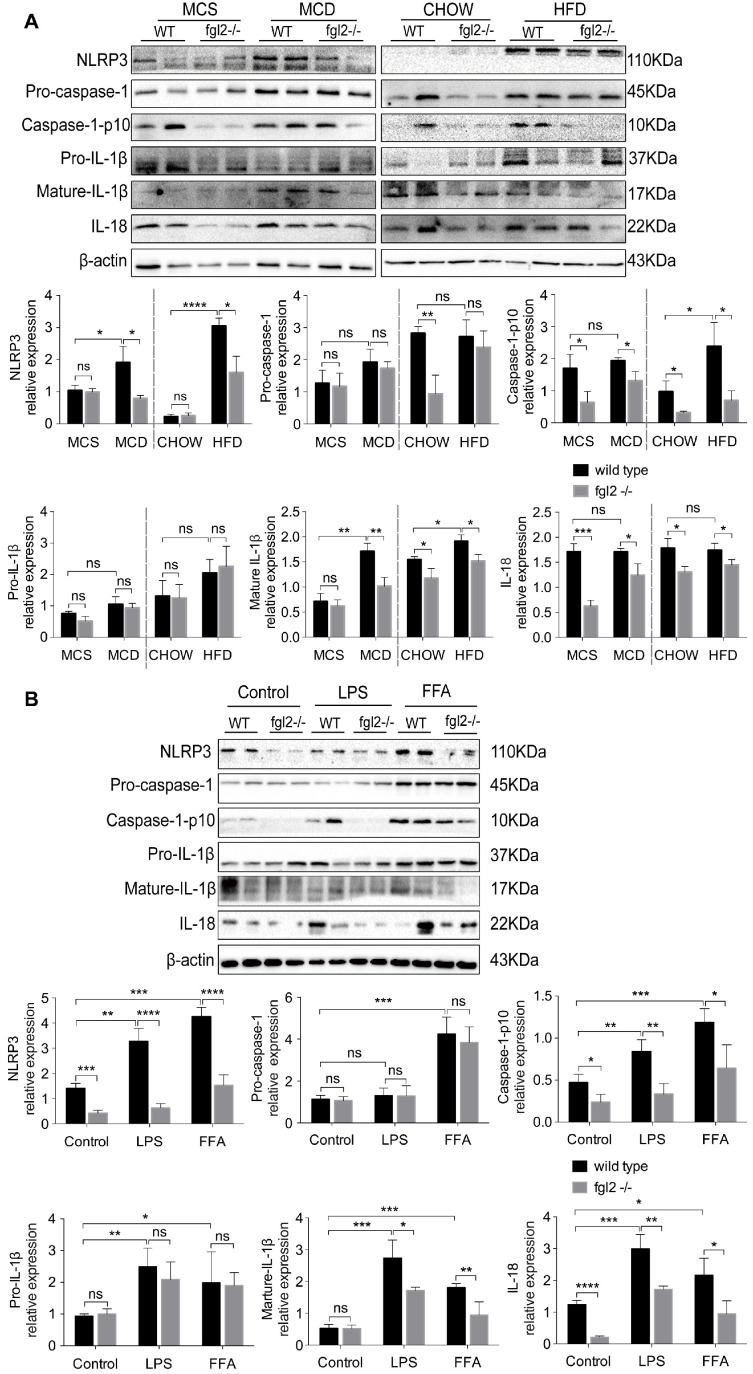
** Fgl2 disruption inhibited activation of the NLRP3 inflammasome in NASH.** Total protein was obtained from liver tissues of MCD-fed or HFD-fed WT and fgl2-/- mice. MCS-fed and chow-fed mice were used as controls. NLRP3, pro-caspase-1, cleaved caspase-1 (caspase-1 p10), pro-IL-1β, mature IL-1β and IL-18 were analyzed by western blotting (A). BMDMs from WT and fgl2-/- mice were stimulated with LPS or FFA and tested for inflammasomes by western blotting (B). Image density was quantified using ImageLab software. For bar graphs, n=6 in each group. The data represent the mean ± SD from at least three independent experiments. Statistical differences were determined by two-way ANOVA. *P<0.05, **P<0.01, ***P<0.001, ****P<0.0001; ns, not significant.

Evidence has been presented that inflammasome activation occurs in NAFLD, and increased expression of inflammasome components could advance NAFLD to NASH 35. Based on our findings, IL-1β and IL-18, the major products of inflammasome activation, were significantly elevated in NASH and were suppressed by fgl2 disruption. Thus, we detected the activation of the NLRP3 inflammasome in the diet-induced fgl2-/- and WT NASH models. In our study, the levels of NLRP3, cleaved caspase-1 (caspase-1 p10), mature IL-1β and IL-18 were all increased in MCD-fed and HFD-fed WT mice compared with controls, while the expression of these proteins was suppressed in fgl2-/-NASH models (Figure 7A). We did not find notable changes in pro-caspase-1 and pro-IL-1β after fgl2 disruption in the MCD or HFD groups (Figure 7A). The results from the *in vitro* experiment were consistent with the findings *in vivo*. Under LPS or FFA stimulation, the levels of NLRP3, caspase-1 p10, mature IL-1β and IL-18 were all lower in fgl2-/- BMDMs than in WT BMDMs (Figure 7B). Pro-caspase-1 and pro-IL-1β, similar to the *in vivo* findings, showed no significant difference. Collectively, these data suggest that inflammasome activation also plays an important role in the mechanism by which fgl2 aggravates liver injury.

### Fgl2 interacted with TLR4 on macrophages and activated MyD88-dependent signaling in NASH

**Figure 8:**
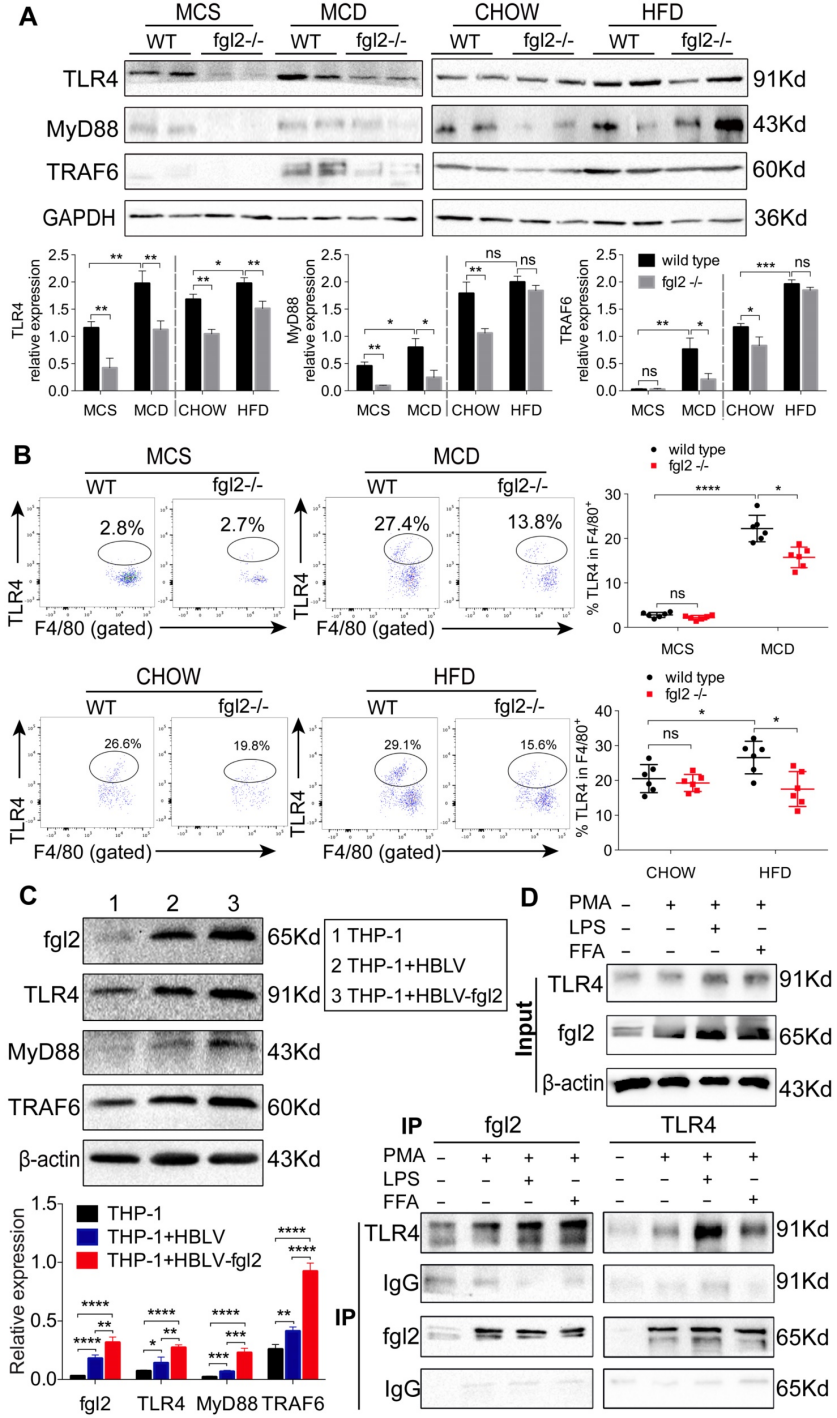
** Fgl2 interacted with TLR4 on macrophages and activated the MyD88-dependent signaling pathway in NASH.** The expression of TLR4, MyD88 and TRAF6 in liver tissues was tested by western blotting in MCD-fed or HFD-fed WT and fgl2-/- mice. MCS-fed and chow-fed mice were used as controls (A). The expression of TLR4 on F4/80+ hepatic macrophages was detected by flow cytometry (B). Fgl2 was overexpressed in THP-1 cells by infection with HBLV-h-Fgl2-GFP-PURO. HBLV-GFP-PURO was used as a control. Then, the expression of fgl2, TLR4, MyD88 and TRAF6 was detected (C). Coimmunoprecipitation (CoIP) of endogenous TLR4 and fgl2 was performed in differentiated THP-1 cells stimulated by LPS or FFA. Protein extracts were immunoprecipitated with an antibody against fgl2 or TLR4, followed by immunoblotting with the indicated antibodies (bottom panel, D). Fgl2 and TLR4 were also detected in total cell lysates (top panel, D). For bar graphs, n=6 in each group. The data represent the mean ± SD from at least three independent experiments. Statistical differences were determined by one-way ANOVA with Bonferroni correction or two-way ANOVA. *P<0.05, **P<0.01, ***P<0.001, ****P<0.0001; ns, not significant.

Increasing evidence suggests that TLR4 signaling plays an essential role in the pathogenesis of NAFLD 8, 36. It is also well established that TLR4 recruits MyD88 to trigger the initial activation of NF-κB and MAPK 37. Our findings indicated that fgl2 disruption blocked the NF-κB and p38-MAPK pathways; thus, we hypothesized that there might be a connection between fgl2 and TLR4. We found that TLR4 expression in liver tissues and hepatic macrophages was significantly decreased upon fgl2 disruption in diet-induced NASH mice compared with WT NASH mice (Figure 8A, B). In addition, the downstream molecules MyD88 and TRAF6 in the liver also showed evident decreases in MCD-fed fgl2-/- mice (Figure 8A).

We then attempted to determine whether fgl2 could directly interact with TLR4 on macrophages. We observed that lentivirus-mediated overexpression of fgl2 in THP-1 cells resulted in higher expression of TLR4, activated downstream MyD88 and TRAF6 (Figure 8C), and upregulated expression of TNF-α, IL-1β and IL-6 (Figure S8A, B, C). Then, we subjected lysates of LPS- or FFA-stimulated THP-1 cells to immunoprecipitation with an antibody against fgl2 or TLR4. The pull-down assay showed that fgl2 directly bound to TLR4 (Figure 8D). From these data, we considered that fgl2 interacted with TLR4 on macrophages and activated MyD88-dependent signaling pathways to induce the production of proinflammatory cytokines and ultimately led to liver injury.

## Discussion

Fgl2 is considered a critical immune regulator in infections 18, 38-40 and cancer progression 41, 42. Fgl2 can be triggered by distinctive signaling pathways, including viral proteins and immune cytokines, especially within the monocyte-macrophage lineage. The outcome and severity of viral hepatitis is closely related to the activity of mfgl2 in macrophages, which leads to the deposition of fibrin and consequent liver necrosis 25. However, the role of fgl2 in NASH is not well understood. The only available data indicated that plasma fgl2 levels in patients with NASH are significantly higher than those in healthy controls 26. In our study, fgl2 expression in the livers of both humans and mice with NASH was markedly enhanced concomitantly with the accumulation of hepatic macrophages. Hepatic macrophages, including resident Kupffer cells and infiltrated monocyte-derived macrophages, are key players in liver innate immunity 43. In different stages of liver disease, hepatic macrophages play different roles in the regulation of inflammation, fibrogenesis and disturbance of lipid metabolism 10. A recent study demonstrated that fgl2 deficiency decreased macrophage infiltration and shifted the macrophage phenotype from proinflammatory (M1) to anti-inflammatory (M2) in early-stage coronary microvascular obstruction (CMVO) 27. In NASH models, we also observed that fgl2 deletion reduced macrophage accumulation in the liver and decreased the production of proinflammatory cytokines and ROS in macrophages. Moreover, adoptive transfer of WT BMDMs into macrophage-depleted NASH mice led to more severe liver inflammation than that in mice transplanted with fgl2-/- BMDMs. This result further suggested the specific role of macrophage-expressed fgl2 in inducing liver inflammatory injury in NASH. It is likely that constitutive expression of fgl2 on macrophages played an essential role in regulating the immune response by supporting M1 phenotype activation and contributed to the progression of NASH. In addition to the alteration of hepatic fgl2, serum fgl2 also increased in MCD-fed mice but decreased in HFD-fed mice (Figure S2D). It is plausible that the excessive hepatic inflammation in MCD-fed mice promoted the transcription and translation of the fgl2 gene, which induced the expression of both mfgl2 and sfgl2. However, in HFD-fed mice, increased oxidative stress causes apoptosis of Tregs and reduces the number of Tregs 44, resulting in reduced sfgl2 expression.

In addition to its influence on inflammation, fgl2 disruption also reduced weight gain, attenuated liver steatosis and insulin resistance in HFD- but not MCD-fed NASH mice. These changes suggested that fgl2 deficiency affected not only the liver but also have major systemic effects in HFD-induced NASH. Although fgl2 deficiency ameliorated liver injury in the MCD model, it could not counteract the effect of MCD diet-induced weight loss in mice. Further study demonstrated that fgl2 participated in modulating hepatic lipid metabolism, displayed as downregulation of lipogenic genes (SREBP-2, Fasn) and upregulation of lipolytic genes (PPARα, CPT1A) by fgl2 disruption. SREBP-2 45, 46 and Fasn 47 induce the accumulation of cholesterol 45 and triglyceride 48 in hepatocytes and directly lead to lipid toxicity. In contrast, PPARα induces the expression of the downstream molecule CPT1A to promote β-oxidation and decreases Fasn expression to inhibit lipid deposition 49. In contrast, incubation of fgl2-deficient BMDM-CM, which contains lower levels of proinflammatory cytokines and ROS than WT BMDM-CM, led to a significant decrease in fat deposition and a beneficial alteration of lipid metabolism in hepatocytes. In fact, increasing evidence has suggested that proinflammatory cytokines and ROS mediate lipogenesis in hepatocytes and aggravate HFD-induced steatosis. ROS increase the expression of SREBP-2 for cholesterol synthesis in the liver when glucose uptake increases 50. Both TNF-α and IL-1β directly inhibit the activation of PPARα 51, 52 to upregulate the expression of Fasn, which combines acetyl-CoA to produce fatty acids 47. TNF-α also suppresses β-oxidation through inhibition of peroxisomal fatty acyl-CoA oxidase 53 to promote hepatic steatosis. IL-6 was found to upregulate the expression of lipogenic enzymes in hepatocytes and lead to steatosis and elevated fat content in the liver 54, 55. Given the concurrent changes in proinflammatory cytokines, ROS and lipid metabolism upon fgl2 disruption, we hypothesize that fgl2 may cause lipid metabolism disorders in the liver through the induction of proinflammatory cytokines and ROS in macrophages. It is worth mentioning that fgl2 may also directly influence lipid metabolism through an unknown pathway that needs further study.

Here, the signaling pathway through which fgl2 triggers the production of proinflammatory cytokines and ROS is still unclear. Many studies have shown the correlation between proinflammatory cytokines, ROS and signaling pathways such as NF-κB and MAPK 32, 56, 57. ROS are proposed to be involved in the activation of NF-κB 57 and p38-MAPK 58, which induces the secretion of TNF-α, IL-1β, IL-6 and MCP-1 59. Since fgl2 induced the production of these cytokines in macrophages, we next explored the correlation of fgl2 with NF-κB and p38-MAPK signaling. We found that fgl2 disruption induced remarkable suppression of NF-κB and p38-MAPK activation in MCD-fed mice, suggesting a regulatory role of fgl2 in these pathways. However, these changes were not observed in HFD-fed mice, which presented much milder inflammation in the liver. In fact, several studies have demonstrated that the NF-κB and p38-MAPK signaling pathways also modulate fgl2 induction and functional activity 60, 61. Thus, we propose that the crosstalk between fgl2 and these two signaling pathways is critical for the overproduction of proinflammatory cytokines and ROS and the subsequent liver damage in NASH. In recent years, another trigger for liver inflammation in NAFLD/NASH has been identified as the activation of inflammasomes 35. Inflammasomes are multiprotein scaffolds that respond to noxious signals (PAMPs, DAMPs) on immune cells and initiate the maturation of the proinflammatory cytokines IL-1β and IL-18 62. Fatty acids 63, 64, cholesterol crystals 65 and ROS 66 are well-characterized inflammasome-activating signals in liver diseases. Our data suggested that fgl2 in NASH models also activated the NLRP3 inflammasome, which facilitated the progression of NASH.

Next, we determined whether there is a link between fgl2 and downstream signaling pathways. Current studies have indicated that Toll-like receptor 4 (TLR4) 8, which is highly expressed on macrophages 67, is a principal receptor for endotoxin, the central mediator of liver inflammation associated with NAFLD 68. The LPS- or FFA-mediated TLR4 signaling pathway activates downstream transcription factors leading to enhanced inflammatory responses, including ROS generation 69, NLRP3 inflammasome-associated IL-1β production 70, NF-κB and MAPK 71 mediated TNF-α 56 and IL-6 production in mouse macrophages and human blood monocytes 72. The above evidence drove us to further explore the potential association between fgl2 and TLR4. Interestingly, in MCD diet-induced NASH mice, fgl2 disruption inhibited the expression of TLR4 and the activation of the downstream MyD88-TRAF6 signaling pathway. *In vitro*, overexpression of fgl2 directly upregulated the expression of TLR4, MyD88 and TRAF6. Furthermore, immunoprecipitation showed a direct combination of fgl2 with TLR4. These data suggested that fgl2 may cooperate with TLR4, activating the MyD88-TRAF6 pathway, which participates in the activation of NF-κB, P38-MAPK and the inflammasome in macrophages. However, in this study, we did not elaborate on the mechanism by which fgl2 interacts with TLR4. Additionally, as a transmembrane protein, it is still unknown whether fgl2 could influence other signaling pathways through its intracellular domain. Additional studies are needed to clarify these questions.

## Conclusions

**Figure 9:**
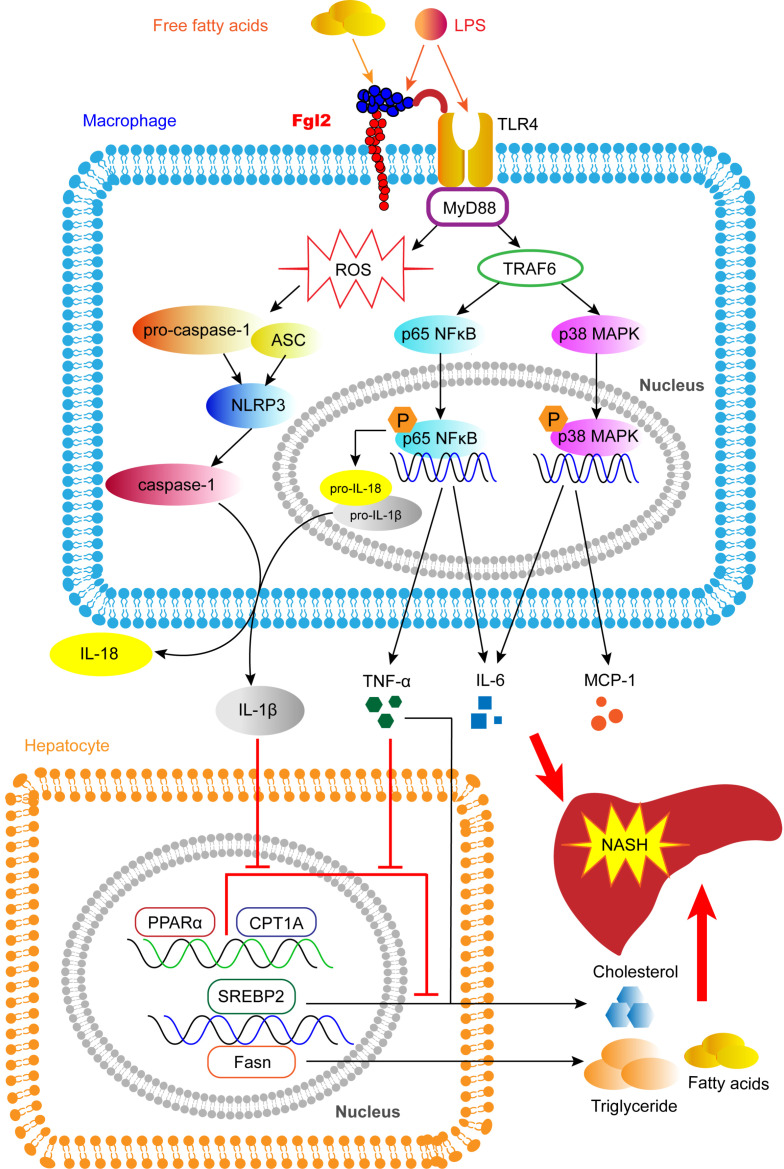
** Fgl2 contributes to the progression of NASH.** In macrophages stressed by LPS, FFA or other stimulators in NAFLD, fgl2 induces ROS production and activates NF-κB and p38-MAPK signaling pathways and the NLRP3 inflammasome. An interaction of fgl2 and TLR4 and subsequent activation of the TLR4-MyD88-TRAF6 axis may be involved in this process. Proinflammatory cytokines such as TNF-α, IL-6, MCP-1, IL-1β and IL-18 are secreted to induce liver injury and lipid metabolism disorders. TNF-α and IL-6 promote the expression of SREBP-2 or Fasn, which are involved in the synthesis of cholesterol, triglycerides and fatty acids. TNF-α and IL-1β downregulate the expression of PPARα and CPT1A, which contribute to lipolysis. These changes result in more significant liver steatosis and further aggravate hepatic inflammatory injury, leading to the progression of NASH.

In this study, we reveal a novel pathologic mechanism by which fgl2 expressed by macrophages upregulates the NF-κB and p38-MAPK signaling pathways and NLRP3 inflammasome, resulting in overproduction of proinflammatory cytokines and ROS, which leads to hepatic lipid metabolism disorders and severe liver injury in NASH (Figure 9). An interaction between fgl2 and TLR4 and the subsequent activation of the TLR4-MyD88-TRAF6 signaling pathway may in part contribute to this process. Thus, fgl2 might serve as a potential biomarker and therapeutic target in the treatment of NASH.

## Supplementary Material

Supplementary figures and tables.

## Abbreviations

WT: wild-type
Fgl2: fibrinogen-like protein 2
NAFL: nonalcoholic fatty liver
NAFLD: nonalcoholic fatty liver disease
NASH: nonalcoholic steatohepatitis
MCD: methionine choline deficient
HFD: high-fat diet
NAS: NAFLD activity score
TNF-α: tumor necrosis factor-α
IL: interleukin
CM: conditioned media
TG: triglyceride
CHOL: cholesterol
Fasn: fatty acid synthase
SREBP-2: sterol regulatory element binding protein 2
PPARα: peroxisome proliferator-activated receptor α
CPT1A: carnitine palmitoyl transferase 1A
ROS: reactive oxygen species
NF-κB: molecules like nuclear factor-κB
MAPK: mitogen-activated protein kinases
NLRP3: NOD-like receptor protein 3
Caspase-1: cysteinyl aspartate specific proteinase-1
TLR4: toll-like receptor 4
MyD88: myeloid differentiation factor 88
TRAF6: TNF receptor associated factor 6
GTT: glucose tolerance test
ITT: insulin tolerance test
