# Supplementary material for: Fibrinogen-like protein 2 aggravates nonalcoholic steatohepatitis via interaction with TLR4, eliciting inflammation in macrophages and inducing hepatic lipid metabolism disorder
Source: Theranostics. 2020 Aug 1;10(21):9702–20. doi: 10.7150/thno.44297 (PMC7449923; doi:10.7150/thno.44297)
Supplement: Supplementary file 1 — Supplementary figures and tables. [file thnov10p9702s1.pdf]

SUPPLEMENTARY MATERIALS AND METHODS

Measurement of serum parameters

Mice were euthanized, and blood samples were collected. Serum was separated and used for biochemical analyses (ALT, AST, LDH and glucose). The levels of serum insulin were measured using a mouse insulin immunoassay kit (SEKM-0141, Solarbio, China). The levels of serum fgl2 were measured by a mouse FGL2 ELISA kit (437807, BioLegend, USA).

Measurement of liver lipid deposition

Frozen mouse liver sections were stained with oil red O for detection of lipids following standard procedures. The level of liver cholesterol was measured by a total cholesterol assay kit (A111-1-1, Nanjing JianCheng, China). The level of liver triglyceride was measured by a triglyceride assay kit (A110-1-1, Nanjing JianCheng, China).

ROS detection

ROS detection was performed using a ROS Assay Kit (S0033, Beyotime, China) according to the manufacturer's instructions. Briefly, the cells from liver tissues and BMDMs were incubated with 10 μM fluorogenic probe dichlorodihydrofluorescein diacetate (DCFH-DA) in serum-free 1640 medium at 37°C for 20 min, and then the cells were washed twice. BMDMs and hepatic nonparenchymal cells not incubated with fluorogenic probe were used as blank controls. Before detection, the viability of BMDMs was tested by the Cell Counting Kit-8 (CK04-01, Dojindo, Japan). Hepatic nonparenchymal cells were stained with fixable viability dye (eBioscience, 65-0864-14, USA) and tested by flow cytometry. The cellular DCF fluorescence intensity of ROS in BMDMs was detected by a fluorescence microplate reader. ROS levels in total liver and hepatic macrophages were detected by an LSRFortessa cytometer.

Glucose tolerance test (GTT) and insulin tolerance test (ITT)

For GTT, mice were fasted for 16 h. After measuring the baseline blood glucose level via a tail nick using a glucometer, 2.0 g/kg glucose was administered via intraperitoneal injection, glucose and insulin (tested by mouse insulin ELISA kit, SEKM-0141, Solarbio, China) levels were measured 0, 15, 30, 45, 60, 90 and 120 min after glucose injection. For ITT, 4-6 h fasted mice were injected intraperitoneally with recombinant human insulin at 1 U/kg and their blood glucose concentrations were determined 0, 15, 30, 45, 60, 90 and 120 min after insulin injection.

SUPPLEMENTARY FIGURE LEGENDS

Figure S1. The specificity of the fgl2 antibody was verified in the livers of NASH mice

To verify the specificity of the fgl2 antibody, the expression of fgl2 in the livers of MCD-fed or HFD-fed WT and fgl2-/- mice was detected by immunohistochemistry (A, B) and western blotting (C).

Figure S2. The accumulated hepatic macrophages showed increased expression of fgl2 in NASH

Expression of CD68 and fgl2 was detected by immunohistochemistry in liver sections of patients with NAFL or NASH or controls (A, arrows indicate CD68 and fgl2 expression). The expression of F4/80 and fgl2 was tested in the livers of MCD- or HFD-fed mice. MCS- and chow-fed mice were used as controls (B, arrows indicate F4/80 and fgl2 expression). F4/80, Clec4f and fgl2 mRNA levels were also detected in MCD- and HFD-fed mice (C). Soluble fgl2 (sfgl2) was measured from the serum of NASH mouse models and controls (D). Fgl2 expression on hepatic macrophages in NASH mice was examined by flow cytometry (E). N=6 in each group. The data represent the mean ± SD from at least three independent experiments. Statistical differences between two experimental groups were determined by unpaired two-tailed Student's t-test. $^{**}P<0.01$, $^{***}P<0.001$, $^{****}P<0.0001$.

Figure S3. Expression of hepatic macrophages and fgl2 in NASH mice during the feeding course

The expression of F4/80+ macrophages (red) and fgl2 (green) was detected by immunofluorescent staining at weeks 0, 2, 4 and 6 in MCD-fed WT mice (A) and at weeks 0, 8, 16 and 24 in HFD-fed WT mice (B). Five microscopic fields per liver section from 3 animals in each group were counted. The data represent the mean ± SD from at least three independent experiments. Statistical differences were determined by one-way ANOVA with Bonferroni correction. $^{*}P<0.05$, $^{**}P<0.01$, $^{***}P<0.001$, $^{****}P<0.0001$.

Figure S4. Fgl2 disruption influenced body weight, glucose tolerance and insulin tolerance in mice

Body weights of MCD-fed mice were detected every week until week 6 (A), while body weights of HFD-fed mice were detected every 2 weeks until week 24 (B). MCS- and chow-fed mice were used as controls (A, B). A, B). Glucose tolerance test (including levels of blood glucose and serum insulin) (C) and insulin tolerance test (D) were performed in WT and fgl2-/- mice. For measurement of body weights, n=10 in each group. For glucose and insulin tolerance tests, n=5 in each group. Statistical differences were determined by two-way ANOVA, and differences between two experimental groups were determined by unpaired two-tailed Student's t-test. *P<0.05, **P<0.01.

Figure S5. Fgl2 expressed by macrophages contributed to the development of NASH

Macrophages were depleted in mice before experiment. Effectivity of macrophage depletion was evaluated by immunohistochemistry (A). NAFLD activity score (B), liver enzymes (C) and proinflammatory cytokines (D) were detected in WT MCD-fed mice that had been transplanted with WT or fgl2-/- BMDMs. Vehicle injection was used as control. For bar graphs, n=6 in each group. The data represent the mean ± SD from at least three independent experiments. Statistical differences were determined by one-way ANOVA with Bonferroni correction. *P<0.05, **P<0.01, ***P<0.001, ****P<0.0001; ns, not significant.

Figure S6. Fgl2 disruption inhibited ROS production in the liver and hepatic macrophages in NASH mice

The ROS levels in the whole liver (A, C) and hepatic macrophages (B, D) from MCD- or HFD-fed WT and fgl2-/- mice were tested by the ROS Assay Kit. MCS- and chow-fed mice were used as controls. BMDMs from WT and fgl2-/- mice were stimulated with LPS (100 ng/ml) or FFA (800 μmol/L). The ROS level in BMDMs was also tested by the ROS Assay Kit (E). N=6 in each group. The data represent the mean ± SD from at least three independent experiments. Statistical differences were determined by two-way ANOVA. *P<0.05, **P<0.01, ***P<0.001, ****P<0.0001; ns, not significant.

Figure S7. Fgl2 deficiency had no significant influence on c-Jun N-terminal kinase activation

Total protein was obtained from liver tissues of MCD-fed or HFD-fed WT and fgl2-/- mice. MCS-fed and chow-fed mice were used as controls. JNK and phosphorylated JNK were analyzed by western blotting (A). Image density was quantified and statistically analyzed using ImageLab software (B). For bar graphs, n=6 in each group. The data represent the mean ± SD from at least three independent experiments. Statistical differences were determined by two-way ANOVA. **P<0.01, ***P<0.001; ns, not significant.

Figure S8. Overexpression of fgl2 promoted the production of proinflammatory cytokines in THP-1 cells

THP-1 cells were infected with HBLV-h-Fgl2-GFP-PURO to overexpress fgl2. THP-1 cells infected with HBLV-GFP-PURO were used as controls. TNF-α (A), IL-1β (B) and IL-6 (C) in cell supernatants were tested by ELISA. N=6 in each group. The data represent the mean ± SD from at least three independent experiments. Statistical differences were determined by one-way ANOVA with Bonferroni correction. *P<0.05, **P<0.01, ***P<0.001.

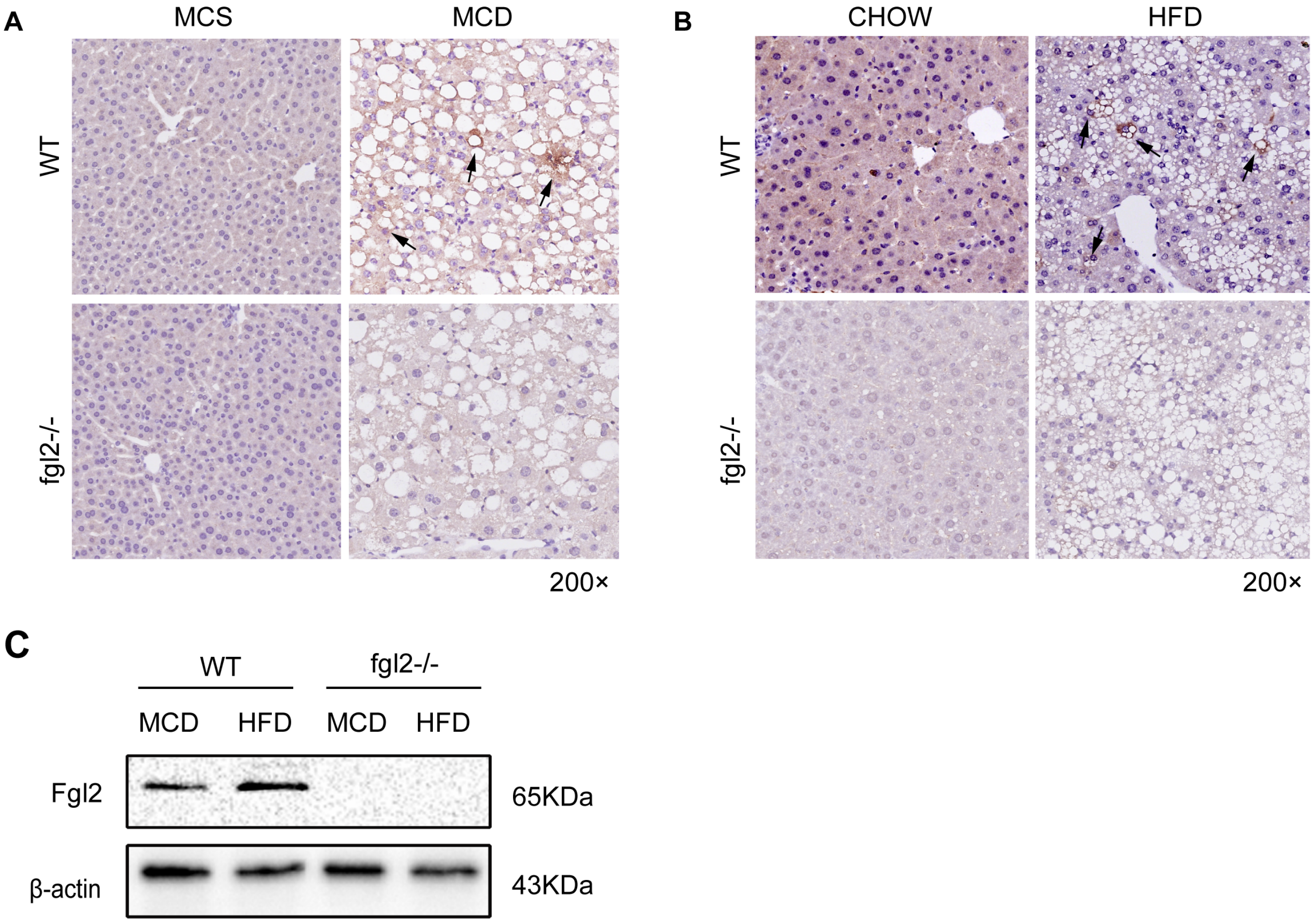
A
MCS
MCD
WT
fgl2-/-
200×
B
CHOW
HFD
WT
fgl2-/-
200×
C
WT
fgl2-/-
MCD
HFD
MCD
HFD
Fgl2
65KDa
β-actin
43KDa

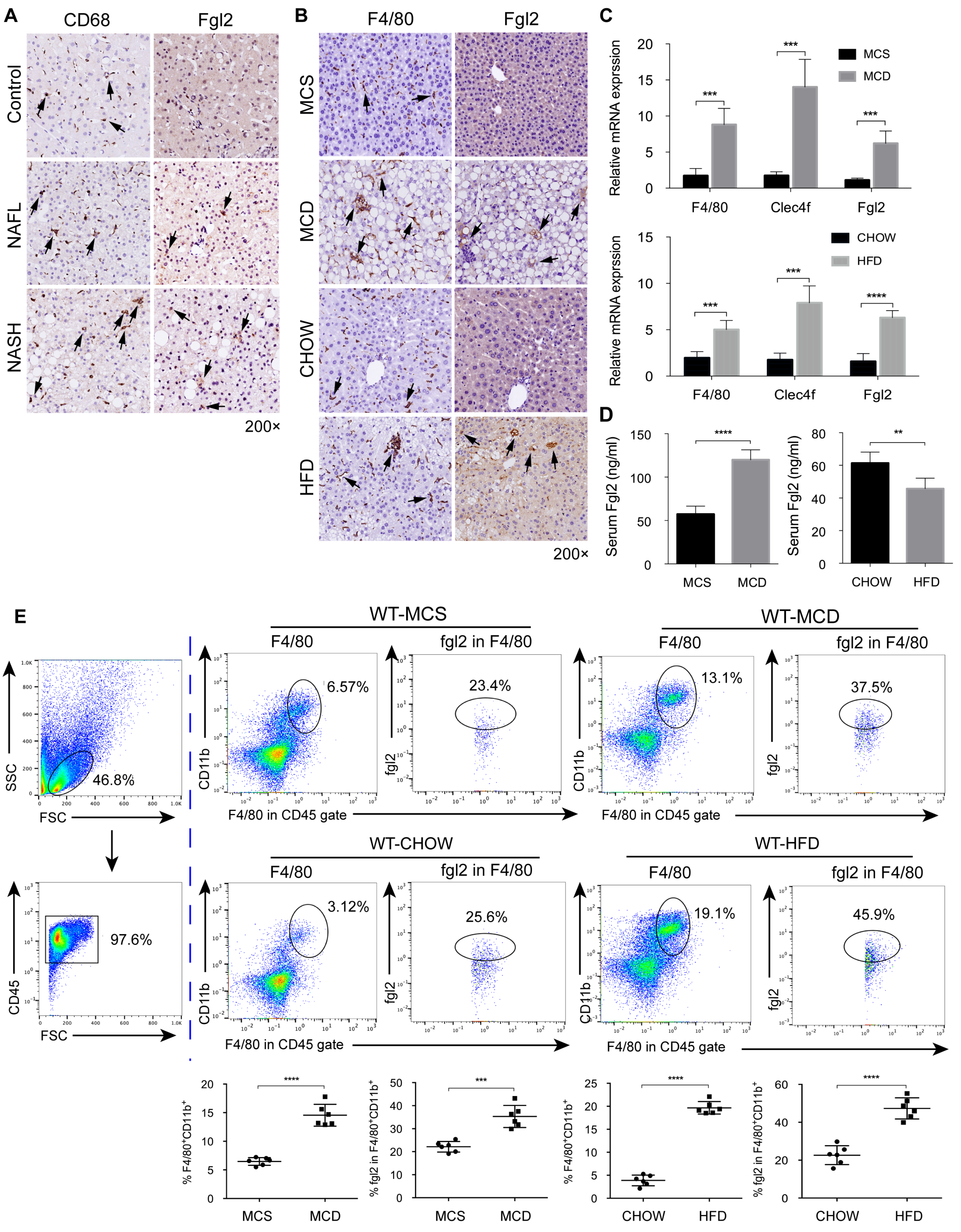

A
CD68
Fgl2
Control
NAFL
NASH
200×
B
F4/80
Fgl2
MCS
MCD
CHOW
HFD
200×
C
Relative mRNA exprssion
MCS
MCD
***
F4/80
Clec4f
Fgl2
CHOW
HFD
****
D
Serum Fgl2 (ng/ml)
**
E
WT-MCS
WT-MCD
WT-CHOW
WT-HFD
F4/80
fgl2 in F4/80
SSC
FSC
46.8%
CD45
97.6%
CD11b
fgl2
F4/80 in CD45 gate
6.57%
23.4%
13.1%
37.5%
3.12%
25.6%
19.1%
45.9%
% F4/80+CD11b+
% fgl2 in F4/80+CD11b+

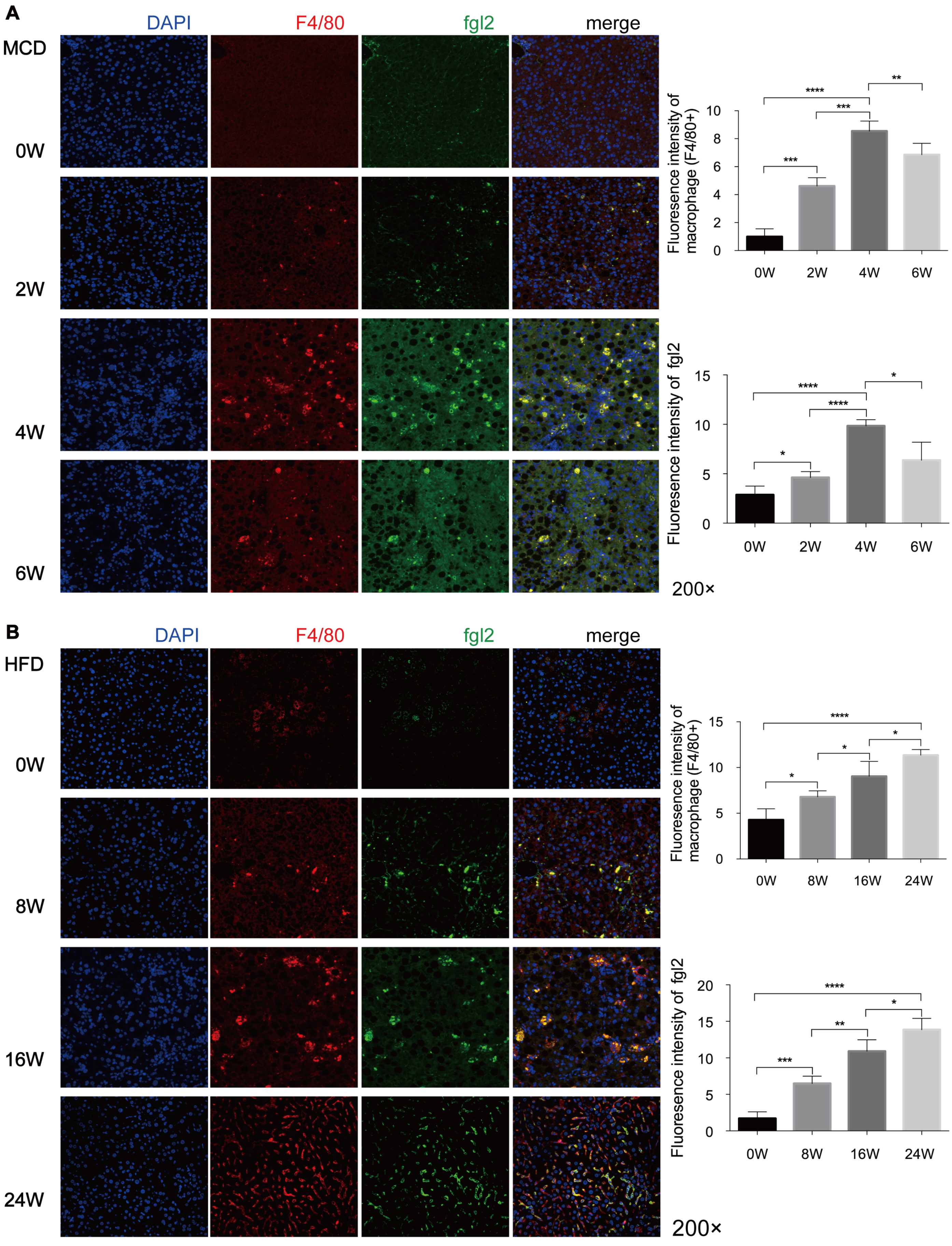
A
DAPI
F4/80
fgl2
merge
MCD
0W
2W
4W
6W
Fluoresence intensity of macrophage (F4/80+)
10
8
6
4
2
0
****
***
**
***
0W
2W
4W
6W
Fluoresence intensity of fgl2
15
10
5
0
****
****
*
*
0W
2W
4W
6W
200×
B
DAPI
F4/80
fgl2
merge
HFD
0W
8W
16W
24W
Fluoresence intensity of macrophage (F4/80+)
15
10
5
0
****
*
*
*
0W
8W
16W
24W
Fluoresence intensity of fgl2
20
15
10
5
0
****
*
**
***
0W
8W
16W
24W
200×

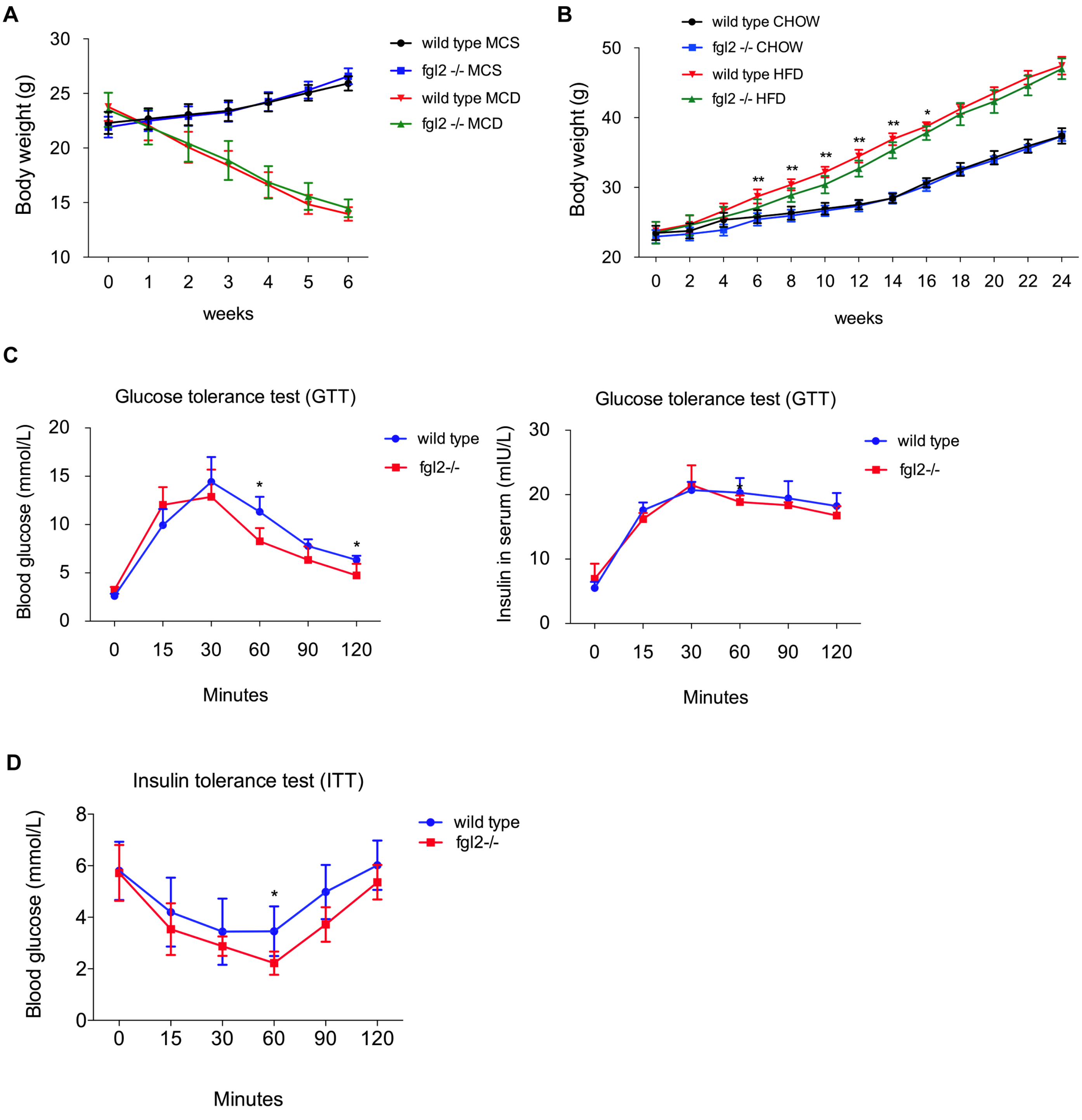
A
Body weight (g)
weeks
wild type MCS
fgl2 -/- MCS
wild type MCD
fgl2 -/- MCD
B
Body weight (g)
weeks
wild type CHOW
fgl2 -/- CHOW
wild type HFD
fgl2 -/- HFD
C
Glucose tolerance test (GTT)
Blood glucose (mmol/L)
Minutes
wild type
fgl2-/-
Glucose tolerance test (GTT)
Insulin in serum (mIU/L)
Minutes
wild type
fgl2-/-
D
Insulin tolerance test (ITT)
Blood glucose (mmol/L)
Minutes
wild type
fgl2-/-

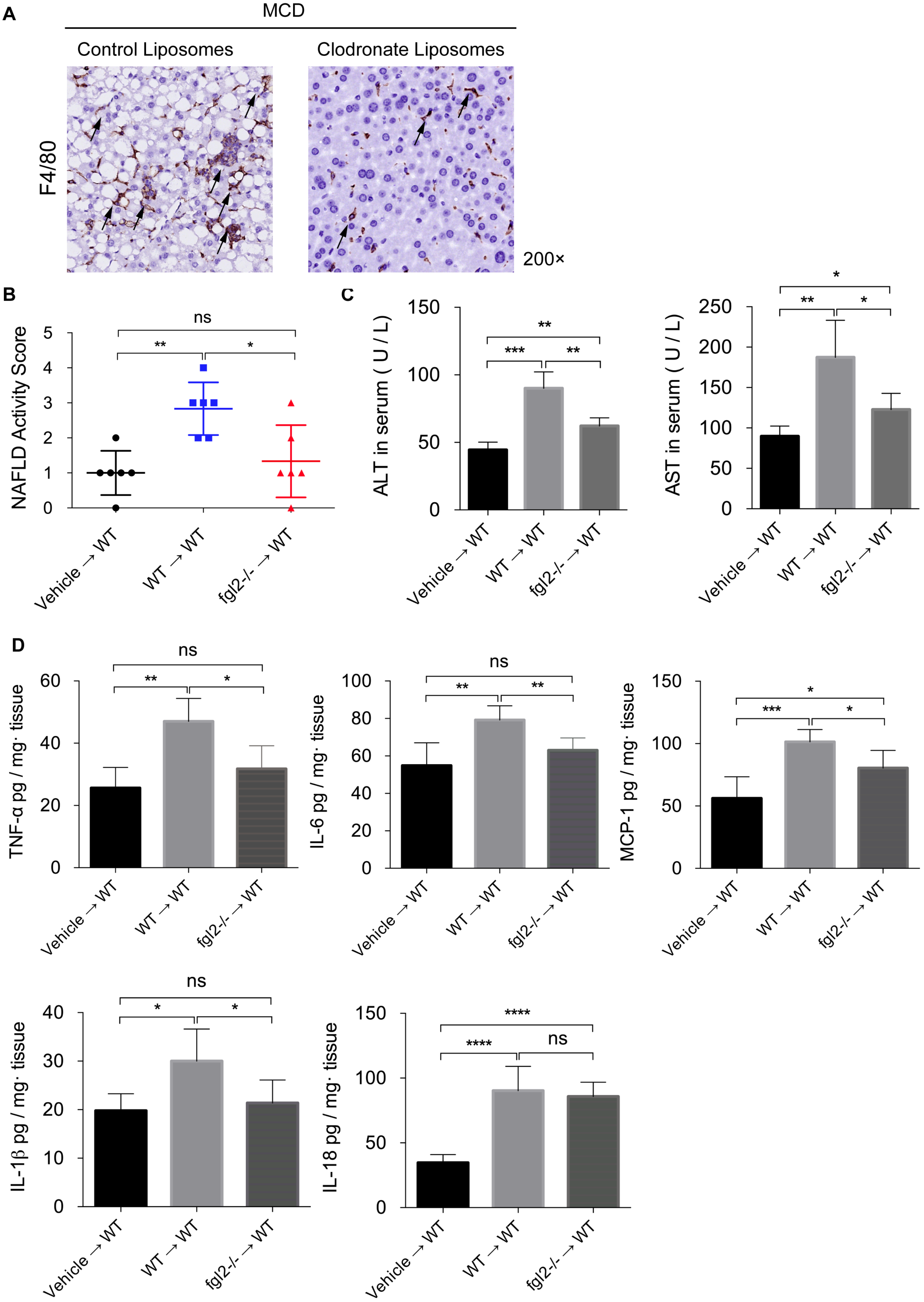

A
MCD
Control Liposomes
Clodronate Liposomes
F4/80
200×
B
NAFLD Activity Score
ns
**
*
Vehicle → WT
WT → WT
fgl2-/- → WT
C
ALT in serum (U / L)
AST in serum (U / L)
***
**
*
D
TNF-α pg / mg· tissue
IL-6 pg / mg· tissue
MCP-1 pg / mg· tissue
IL-1β pg / mg· tissue
IL-18 pg / mg· tissue
****

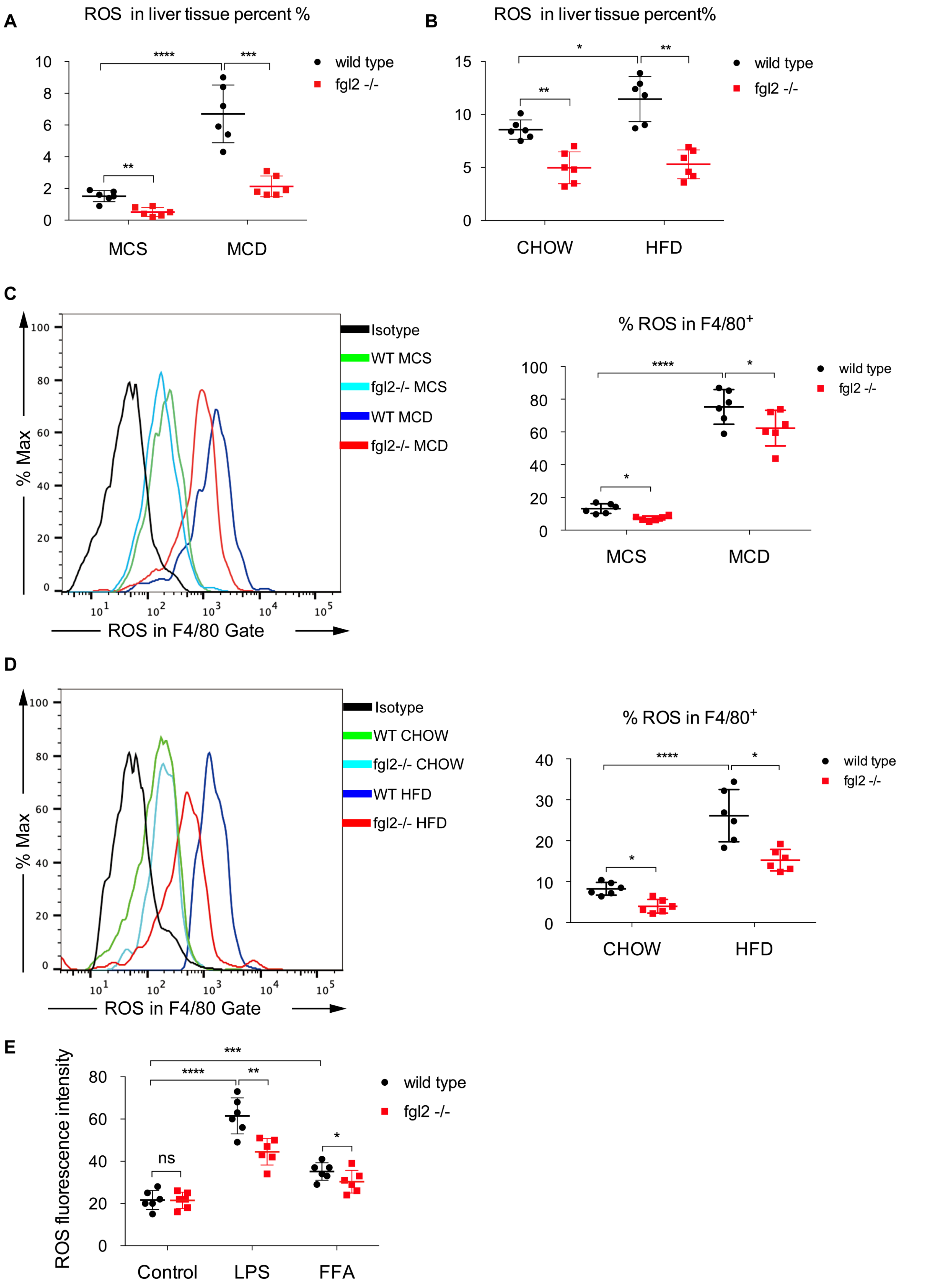
A
ROS in liver tissue percent %
wild type
fgl2 -/-
MCS
MCD
B
ROS in liver tissue percent%
wild type
fgl2 -/-
CHOW
HFD
C
Isotype
WT MCS
fgl2-/- MCS
WT MCD
fgl2-/- MCD
% Max
ROS in F4/80 Gate
% ROS in F4/80+
wild type
fgl2 -/-
MCS
MCD
D
Isotype
WT CHOW
fgl2-/- CHOW
WT HFD
fgl2-/- HFD
% Max
ROS in F4/80 Gate
% ROS in F4/80+
wild type
fgl2 -/-
CHOW
HFD
E
ROS fluorescence intensity
wild type
fgl2 -/-
ns
Control
LPS
FFA

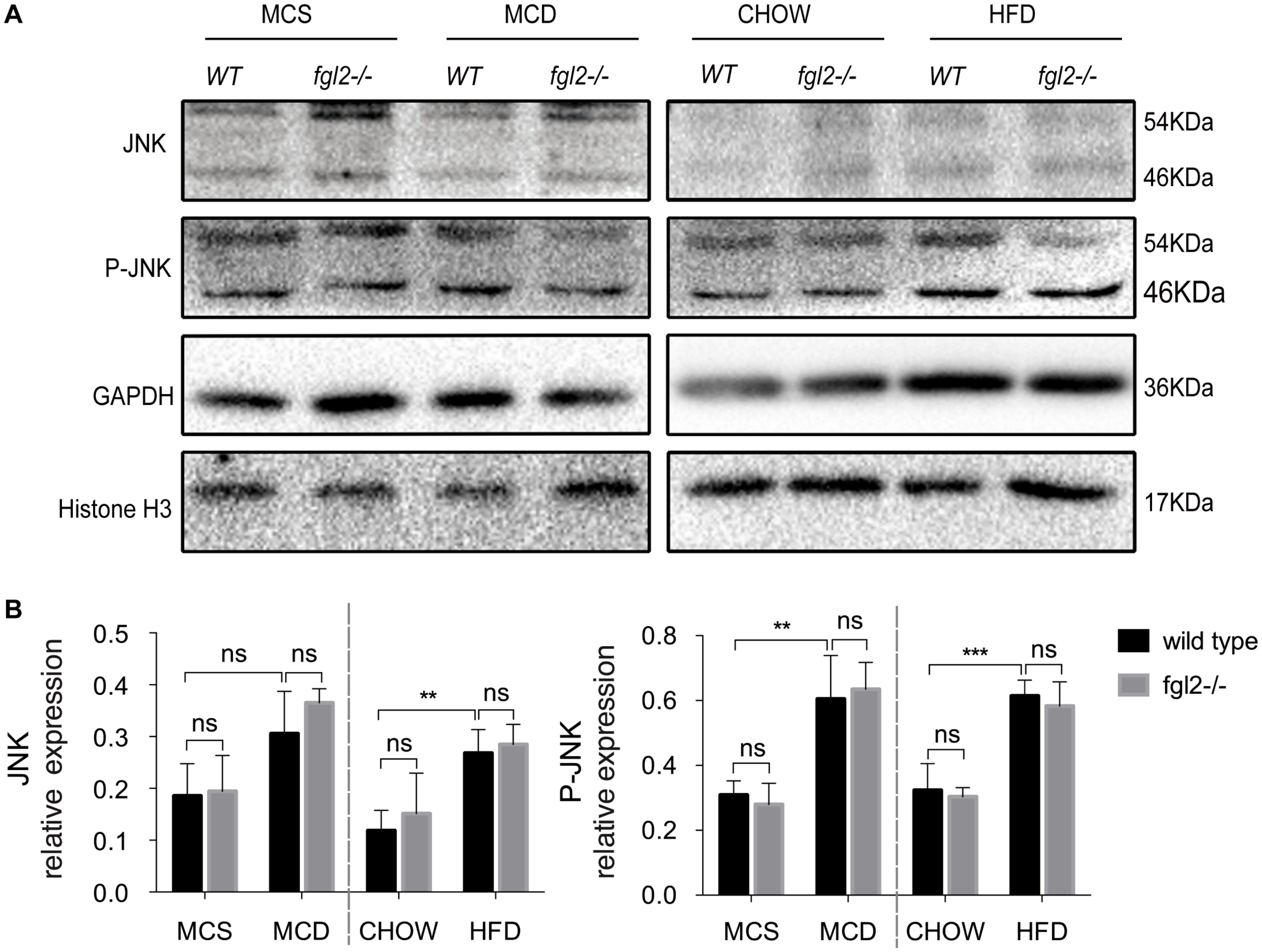

A
MCS
MCD
CHOW
HFD
WT
fgl2-/-
WT
fgl2-/-
WT
fgl2-/-
WT
fgl2-/-
JNK
P-JNK
GAPDH
Histone H3
54KDa
46KDa
54KDa
46KDa
36KDa
17KDa
B
JNK relative expression
P-JNK relative expression
0.5
0.4
0.3
0.2
0.1
0.0
0.8
0.6
0.4
0.2
0.0
ns
**
***
MCS
MCD
CHOW
HFD
wild type
fgl2-/-

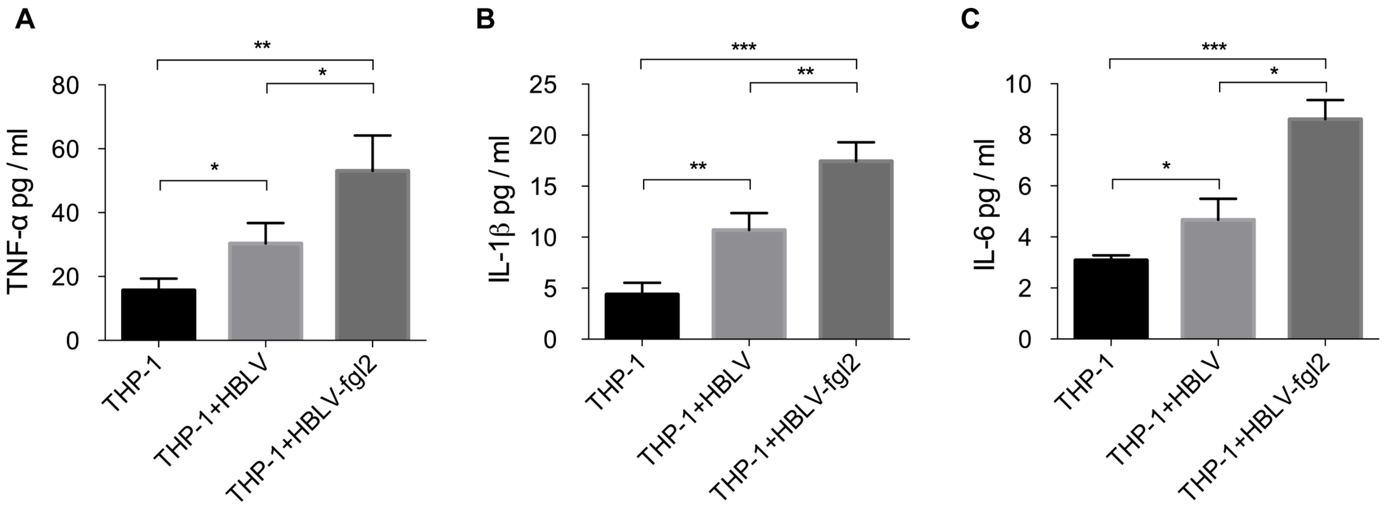
A
B
C
TNF-α pg / ml
IL-1β pg / ml
IL-6 pg / ml
0
20
40
60
80
0
5
10
15
20
25
0
2
4
6
8
10
*
**
***
THP-1
THP-1+HBLV
THP-1+HBLV-fgl2

Table S1. Characteristics of the human subjects

	Control	NAFL	NASH
**Subject number**	6	6	8
**Gender, male**	4 (66.7%)	3 (50%)	5 (62.5%)
**Age (years)**	54.3 ± 9.0	47.3 ± 8.4	38.1 ± 12.0
**ALT (IU/L)**	17.8 ± 5.8	16.7 ± 7.0	113.1 ± 39.2 (*P <0.001, #P <0.001)
**AST (IU/L)**	19.1 ± 2.1	17.5 ± 4.0	61.3 ± 22.1 (*P <0.01, #P <0.01)
**NAFLD activity score (n)**	0 (3) 1 (3)	3 (2) 4 (4)	5 (1) 6 (3) 7 (4)
Grade of steatosis (n)	0 (3)	1 (6)	2 (3) 3 (5)
Lobular inflammation (n)	1 (3)	1 (5)	1 (1) 2 (7)
Hepatocellular ballooning (n)	0 (3)	1 (3) 2 (4)	1 (1) 2 (7)

NAFL and NASH patients were diagnosed by two board-certified pathologists (Dr. Yonggang Liu and Dr. Ruifang Shi) and evaluated with NAFLD activity score. The controls were human subjects with normal liver histology. Data are presented as mean ± SD and statistical difference was determined by Mann-Whitney U test. *P compared with "Control", #P compared with "NAFL".

Table S2. Methionine choline deficient diet composition

Components	g/kg	Components	g/kg
Sucrose	455.3	L-Isoleucine	8.2
Corn Starch	200.0	L-Leucine	11.1
Corn Oil	100.0	L-Lysine Hydrochloride	18.0
Alphacel Non-Nutritive Bulk	30.0	L-Phenylalanine	7.5
AIN 76 Mineral Mix	35.0	L-Proline	3.5
Dicalcium Phosphate	3.0	L-Serine	3.5
L-Alanine	3.0	L-Threonine	8.2
L-Arginine Hydrochloride	12.1	L-Tryptophan	1.8
L-Asparagine Monohydrate	6.0	L-Tyrosine	5.0
L-Aspartic Acid	3.5	L-Valine	8.2
L-Cystine	3.5	DL-alpha-Tocopherol Acetate (250 u/g)	0.484
L-Glutamic Acid	40.0	Vitamin A Palmitate (250,000 u/g)	0.0792
Glycine	23.3	Vitamin D3 (400,000 u/g)	0.0055
L-Histidine Hydrochloride	4.5	Ethoxyquin	0.02

Table S3. High fat diet composition

Components	g/kg	Components	g/kg
Casein	195	Mineral Mix, M1021	68
Maltodextrin	225	Vitamin Mix, V1010	14
Sucrose	89	L-Cystine	3
Soybean Oil	33	Choline Bitartrate	3
Lard	301	TBHQ	0.067
Cellulose	69	Cholesterol	0.217

Total calories: 5 kcal/g (Protein: 14.1%, Carbohydrate: 25.9%, Fat: 60.0%)
